# Exposure to conflict and child health outcomes: evidence from a large multi-country study

**DOI:** 10.1186/s13031-022-00483-9

**Published:** 2022-10-10

**Authors:** Srinivas Goli, Astghik Mavisakalyan, Anu Rammohan, Loan Vu

**Affiliations:** 1 Australia India Institute, University of Western Australia, Perth, Australia; 2 Bankwest Curtin Economics Centre, Curtin University, Perth, Australia; 3 Department of Economics, University of Western Australia, Perth, Australia; 4 Department of Fertility and Social Demography, International Institute for Population Sciences (IIPS), Mumbai, India

**Keywords:** Conflict, Child nutrition, Immunization, Multi-country analysis

## Abstract

**Background:**

Previous research has consistently found evidence of poor health outcomes among children living in conflict areas. However, the methodological focus of these studies has largely been on case studies, chart or registry reviews, qualitative studies, and single country studies. This reflects the need for a comprehensive multi-country analysis of the associations between conflicts and child health over a longer period. This study analyses the adverse impact of exposure to different types of conflicts  from in utero to five years of age, on several child health measures across a large group of countries. Our analysis pools data from multiple countries and time-points, to provide robust evidence on the relationship between conflict and child health.

**Methods:**

Geo-referenced data on various forms of conflict are combined with the *Demographic Health Survey* dataset, to construct a large unique database of 590,488 pre-school age children across 52 developing countries over the period 1997 to 2018. Our analysis exploits the within-country differences in children’s exposure to conflict from in utero to age five, to estimate its association with health outcomes. Our multivariate regression models estimate the links between conflict exposure and child health outcomes, measured using child nutrition outcomes (height-for-age and weight-for-age z-scores) and immunization status.

**Results and conclusions:**

Empirical estimates show that even after controlling for a large array of socio-economic and demographic characteristics and location fixed effects, conflict exposure is negatively associated with child nutrition and immunization, across all our measures of conflict. These findings are robust across a range of specifications, alternative measures of conflict and sub-samples.

**Supplementary Information:**

The online version contains supplementary material available at 10.1186/s13031-022-00483-9.

## Introduction

About one in ten children worldwide are affected by armed conflict, with an estimated 246 million children living in conflict-affected areas [1, 2]. Previous research has consistently found evidence of poor health outcomes among children living in conflict areas. Both civil and armed conflicts affect child health outcomes through several different mechanisms starting from in utero, leading to adverse effects on birth weight [3–5], height-for-age [6, 7], weight-for-height [6, 8] and immunization rates [9–13].

**Fig. 1:**
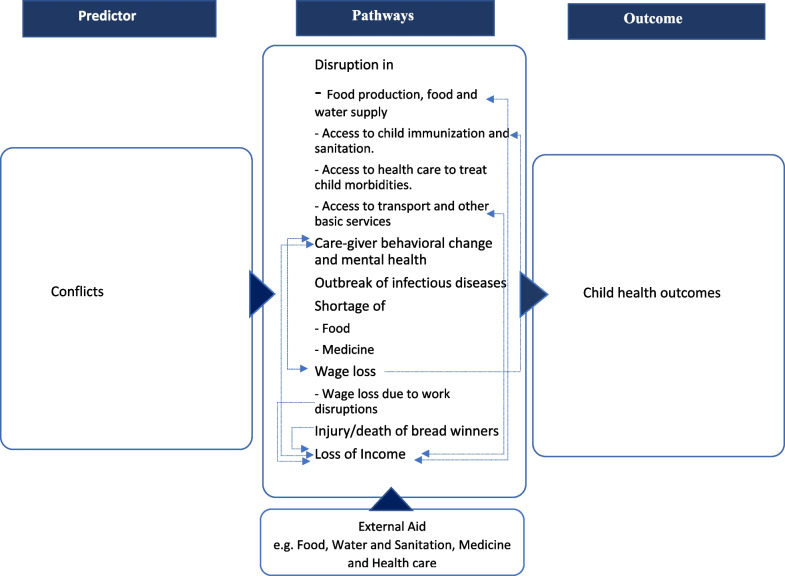
Conflict and health: mechanisms

Conflict exposure can adversely influence population health, both directly and indirectly, through the destruction of health systems, infrastructure, and disruption in services. In the context of child health, some of the underlying factors influencing this relationship include poor access to essential medicine, health care, immunization and basic sanitary services. Conflicts also impact on maternal or caregiver mental health, behavioural changes in response to stress (e.g. dietary intake and diversity, smoking, exercise, and alcohol consumption), selective foetus mortality, pre-term deliveries, unsafe or inadequate living conditions, hunger and chronic insufficient food intake, sustained incorrect feeding practices of babies, frequent infections and diarrheal diseases. Conflicts may also disrupt health infrastructure and cause outbreaks of infectious diseases, disruption in economic activities, wage and income loss and an increase in violence against women [3, 9–11, 14, 15]. Figure 1 presents a path-diagram describing these potential mechanisms through which conflict leads to poor child health outcomes.

In this paper, we empirically estimate the relationship between exposure to conflict from in utero to age 5, and its association with child health outcomes using a sample of 590,488 pre-school age children across 52 countries over the period 1997 to 2018. Our study makes several unique contributions to the literature on conflict and child health. Firstly, using geo-referenced data on three types of violent conflicts (*i.e. state-based, non-state, and one-sided* conflicts) from the *Uppsala Conflict Data Program’s (UCDP)* Georeferenced Event Dataset, we link the location of conflict incidents (both level and severity of conflicts) with mother-children pairs using nationally representative household level data from the *Demographic Health Surveys (DHS),* which use a uniform questionnaire across all countries and over time. This allows us to construct a large unique database to measure the heterogeneous impact of various measures of conflict within different countries over time, and to quantitatively estimate the links between conflict exposure (in utero and during childhood) on child health outcomes.

Secondly, while the literature is unanimous on the negative relationship between conflict and child health measures, the focus of the literature to date has been on single country settings at one point in time, focusing on one particular form of conflict (typically civil war) or one measure of child health. A recent systematic review of studies on conflict and child health found that out of 155 studies to date, the methodological focus has largely been on case studies, chart or registry reviews, qualitative studies. Also, most of them were cross-sectional design studies [1]. This reflects the need for a comprehensive multi-country study. More recently, studies have examined the implications of armed conflict on maternal and child health using aggregate data [14], neo-natal, postnatal and maternal mortality [15]; the delivery of maternal and child health interventions in select countries affected by conflict [16, 17]. Our study differs from these in several important respects. Importantly, while the above studies have used aggregate data from multiple sources, our maternal and health data are based on mother-level surveys that were conducted in over 52 developing countries over the period 1997–2019. Our paper is closest to Jawad and colleagues [14] who also use the same conflict dataset that we use. However, their unit of analysis is at the country-level unlike ours. By focusing on different types of conflicts, and several child health measures across a large group of countries using mother and child-level information, we are able to provide a more nuanced analysis by controlling for a greater array of explanatory variables than is possible using aggregate country-level data. Our analysis pools individual-level data from multiple countries and time-points, to provide robust evidence on the relationship between conflict and child health.

Our child health outcome measures are the two child anthropometric measures of height-for-age (HAZ, a measure of linear growth) and weight-for-age (WAZ, a measure of underweight or overweight) and full immunization status of children. Our most extensive empirical specification is based on comparing children living in the same region of a same country whose exposure to conflict varies due to the length of time they spent under conflict conditions. Therefore, our approach directly controls for key sources of unobserved heterogeneity. Our analysis provides robust evidence that lifetime exposure to conflict is indeed associated with significant deterioration across all three health outcomes for children in the 52 developing countries in our sample.

In “Background and literature review” section, we outline the background and review the relevant literature. This is followed by “Data and methods” section where we present the data and methods. The “Results” section presents the main results from our empirical analysis, and the “Conclusions” section presents the main Conclusions of the study.

## Background and literature review

### Empirical evidence on conflict and child nutrition

The two anthropometric measures of *height-for-age (HAZ) and weight-for-age (WAZ)* are critical measures of child nutrition, and reflect nutritional status over different durations. Underweight (weight-for-age) is determined by short-term energy balance and is therefore an indicator of acute undernutrition, while stunting (height-for-age) is determined by an inadequate energy balance over time and indicates chronic malnourishment. Child nutrition requires adequate feeding and care practices, sanitation, dietary diversity, nutrient density, and a satisfactory level of short- and long-term health. These are difficult to achieve in a conflict environment, and therefore child anthropometric measures provide an objective assessment of the impact of conflict on child health.

Studies on the relationship between civil war and child HAZ unanimously find a statistically significant and negative relationship between conflict exposure and HAZ. However, the focus of this literature has been on ongoing armed civil conflicts. These include studies on the impact of civil conflict on HAZ in Ethiopia [1], Burundi [18] and Cote d’Ivoire [7]. Previous research on the relationship between conflict and child WAZ generally finds a statistically significant, negative relationship between the two variables. Studies from Angola [6], Uganda [19], Nigeria [20] and Afghanistan [21] all found a statistically significant association between conflict and infant WAZ. In the Afghan context, each additional fatality per 10,000 inhabitants during pregnancy caused a 0.20 standard deviation reduction in WAZ [21], and in Angola [22] an additional 100 suspected hazard areas within 150 km of the infant led to a reduction in WAZ by 0.40–0.48 standard deviations depending on the dataset used.

### Empirical evidence on conflict and child immunization

Immunization is a vital and cost-effective disease prevention and control strategy. Despite the growth in vaccine development and immunization delivery systems globally, children in areas of conflict areas often have inadequate or no access to lifesaving vaccines [2, 23]. Country case studies from the BRANCH consortium (*i.e.* Bridging Research & Action in Conflict Settings for the Health of Women and Children) find significant disruptions in immunization coverage of children due to armed and civil conflicts in Angola, Somalia, Yemen, South Sudan, Syria, and Nigeria [24]. A study on the association between armed conflict and vaccination uptake during the Boko Haram insurgency in North Eastern Nigeria found that the odds of a child receiving any vaccination is 47.2 percentage points lower if an armed conflict occurred within 10 km [13].

An assessment from 16 countries finds that the onset of conflict is associated with an unexpected decline in countrywide and sub-national coverage of vaccinations. They attribute this to poor safety, damaged health infrastructure and exhausted human resources which led to infrequent outreach services, and delays in new vaccine introductions and immunization campaigns [10]. Systematic review studies have reported that armed conflicts globally are at an all-time high, and are negatively associated with vaccination coverage [1, 12]. Yet the impact of conflict on global immunization goals has not been fully addressed.

## Data and methods

### Data sources and sample

The aim of this paper is to analyze the association between lifetime conflict exposure and child health in a large sample of developing countries. We combine data from two main sources—the *Uppsala Conflict Data Program* (UCDP) and the *Demographic and Health Surveys* (DHS). Data on conflict is obtained from the UCDP Georeferenced Event Dataset version 19.1. This dataset provides information on the dates of start and termination, locations, and the number of deaths caused by all violent events around the world (excluding Syria) over the period 1989–2018. UCDP defines a violent event as: “an individual incident of lethal violence occurring at a given time and place” [25]. This information is available for three types of organized violence: *state-based violence* (Type 1); *non-state violence* (Type 2); and *one-sided violence* (Type 3). The distinguishing feature of Type 1 violence is the involvement of government as at least one of the two parties between which armed force is used. Type 2 violence, on the other hand, involves the use of armed force between two organized armed groups neither of which is the government. Finally, Type 3 violence involves the use of armed force, by the government or a formally organized group, against civilians. The formal definitions of these three types of violence are presented in Table 1.

**Table 1:** Conflict variables and definitions

*Types of violence*^×^ *categorized by the Uppsala Conflict Data Program (UCDP)*	
Type 1 (State-based conflict)	The use of armed force between two parties, of which at least one is the government of a state
Type 2 (Non-state conflict)	The use of armed force between two organized armed groups, neither of which is the government of a state
Type 3 (One-sided conflict)	The use of armed force by the government of a state or by a formally organized group against civilians
*Constructed measures of conflict for the current study#*	
Conflict1	= 1 if the child was exposed to at least 1 violent event (including all types of violence), 0 otherwise
Conflict2	Number of years between the earliest and the latest violent events (including all types of violence)
Conflict3	Observations with positive deaths per year between the earliest and the latest violence events (including all types of violence) are categorized into 3 terciles from 1 to 3, otherwise 0
Conflict3_cat1	= 1 if Conflict3 = 1, 0 otherwise
Conflict3_cat2	= 1 if Conflict3 = 2, 0 otherwise
Conflict3_cat3	= 1 if Conflict3 = 3, 0 otherwise
Conflict4	= 1 if the child was exposed to at least 1 violent event of Type 1, 0 otherwise
Conflict5	= 1 if the child was exposed to at least 1 violence event of Type 3, 0 otherwise

^×^The term “*violence*” is originally used by the UCDP Georeferenced Event Dataset. Children exposed to violence Type 2 is not included because this type accounts for only 10% of conflict events between 1997 and 2018.

# Variables are constructed accounting for conflict within the child’s province of residence from conception year to the year of the interview

It should be noted that UCDP only records conflicts that have resulted in at least 25 battle-related deaths in a calendar year. This effectively implies that smaller-scale or frozen conflicts, or upheavals that do not necessarily result in deaths but have major political and economic consequences are not captured in the data. In spite of this shortcoming, we choose to use UCDP due to its recognized quality of data collection relative to other sources such as Armed Conflict Location Events Dataset (ACLED) [26].

Data on children’s health outcomes and their family characteristics come from DHS—a collection of nationally-representative repeated cross-sectional surveys conducted in over 90 developing countries since its inception in 1984. DHS interviews women aged 15 to 49 about the birth history of children born in the five years prior to the survey. Surveyors also collect anthropometric measures, socio-economic and demographic characteristics of surveyed women and their children up to the age of 5 years or below. In addition, DHS provides the geo-referenced information of the residential location of households, including the names and GPS coordinates in more recent waves.

**Fig. 2:**
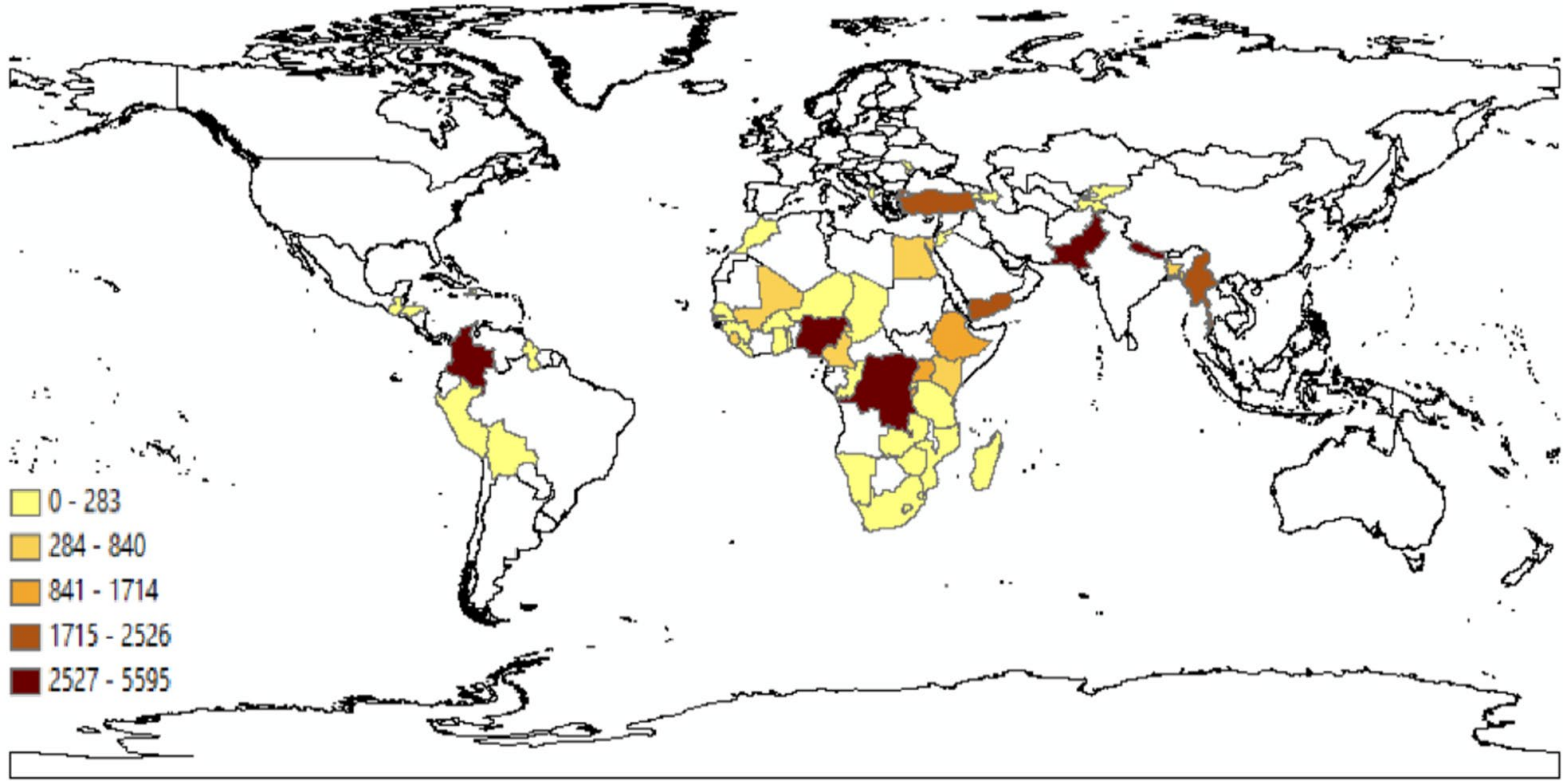
Sample of 52 countries by number of conflict events taking place between 1997 and 2018

Based on the availability of standardized information on key variables of interest across countries and waves, we include only the DHS surveys conducted from 2003 onwards.^1^ Initially, we append the datasets from 68 countries. However, we drop eight countries that are not presented in the UCDP Georeferenced Event Dataset, and seven additional countries with no information on our variables of interest related to characteristics of children, mothers, and households. Moreover, we exclude India from the baseline sample due to its large population size which makes up for one-third of the combined sample.^2^ After excluding outliers in child nutritional status indicators (Z-scores < − 5 or > 5), the final sample includes 590,488 observations from 111 surveys across 52 countries (Fig. 2, Additional file 1: Table S1).

We combine the UCDP and DHS datasets across spatial and temporal dimensions. In the spatial domain, we exploit the names of geographical locations at the level of provinces available in both datasets. In the UCDP data, these are the names of the provinces where a conflict took place. In the DHS dataset, these are the names of the provinces of children’s residence. If there are differences in the names of provinces across the two datasets, we manually reconcile these differences for merging purposes. In total, there are 545 different provinces across the 52 countries in our sample. It should be noted that the definition of provinces varies by country. The averages of provincial-level population by country estimated based on the DHS data are presented in the Additional file 1: Fig. S1.

We do not employ the information on latitude/longitude positions in the datasets mainly for two reasons. Firstly, because the GPS data is available for a much smaller sample of DHS countries; 41 countries have datasets with these variables. Secondly, the GPS coordinates were positioned randomly^3^ in all countries and as such, do not allow us to accurately capture the location of residence for matching purposes. However, we have conducted additional robustness exercises for the sample of countries where geocoded information is available, using a more precise location of survey respondents to define exposure to conflict.

In the temporal domain, we utilize the information on the year when the conflict started and terminated in UCDP. We use the information on the children’s month of birth in the DHS to determine the year of their conception: a child was conceived in the year before their birth year if being born between January-August, otherwise the year of conception coincides with the year of birth. We assign the information on conflict events (including the length of events and associated deaths by conflict type) that have taken place from the year of conception to the year of the interview to each child in each province. The oldest child in our sample was conceived in 1997, and as such, the history of conflict in our analysis dates back to 1997. Given this approach to combining the two datasets, we observe differences in lifetime exposure to conflict among children within and across the 545 provinces employed in the analysis. See Additional file 1: Table S2 for information on conflicts by country and over time.

Finally, we use population data from the World Development Indicators (WDI) to calculate new denormalized sampling weights in the DHS data. The new sampling weights are equal to the interviewed women’s sampling weights multiplied by the fraction between the total number of women aged 15–49 years and the number of women aged 15–49 years interviewed in the DHS in a year for each country.

### Variables

#### Outcome variables

The main outcome variables in this analysis are anthropometric measures of child nutrition that can be directly and indirectly affected by conflict: HAZ and WAZ, and immunization status of children. HAZ measures the child’s linear growth according to age, reflects the cumulative effects of growth deficiency and is used to measure long-term nutrition. WAZ is used to monitor the growth of children and is typically regarded as a measure of short-term nutrition. These anthropometric measures are expressed as Z-scores in standard deviations from the mean of the reference population, calculated using WHO’s current Child Growth Standards reference population median and standard deviation^4^ [27]. It is well-established in the demographic and public health literature that in optimal environments, there are no differences among sampled populations in child growth across geographic, ethnic, and cultural lines. Any deviation from optimal growth may be explained by socio-economic and environmental factors [27, 28]. The DHS provides height, weight, and age of the surveyed children that we use to calculate HAZ and WAZ scores. Furthermore, based on these measures, we also construct additional outcome variables: binary measures for child stunting (HAZ lower than − 2 standard deviations) and underweight (WAZ lower than -2 standard deviations) status. As Table 2 shows, stunting prevalence in the sample is nearly 35%, while 20.5% of the children are underweight.

**Table 2:** Descriptive statistics

Variables	Mean	Std. Dev
*Outcome variables: child health measures*		
Height-for-age Z-score (HAZ)	− 1.354	1.610
Weight-for-age Z-score (WAZ)	− 0.949	1.297
Stunting (HAZ < − 2)	0.349	0.477
Underweight (WAZ < − 2)	0.205	0.403
Immunization (received full set of 8 recommended vaccines)	0.432	0.495
*Explanatory variables: conflict measures*		
Conflict1	0.408	0.491
Conflict2	0.970	1.693
Conflict3		
Conflict3_cat1	0.132	0.338
Conflict3_cat2	0.131	0.338
Conflict3_cat3	0.131	0.338
Conflict4	0.232	0.422
Conflict5	0.271	0.444
*Control variables: characteristics of children, mothers, and households*		
Child's age (months)	29.063	17.193
Child is male	0.505	0.500
Child is multiple birth	0.024	0.153
Birth order number	2.650	1.204
Mother's age (years)	28.594	6.646
Mother's height (cm)	156.186	6.975
Mother’s age at 1st birth	19.571	3.984
Mother’s age at 1st cohabitation	18.460	4.117
Mother is using contraception	0.422	0.494
Mother’s education: primary	0.301	0.459
Mother’s education: secondary	0.287	0.452
Mother’s education: higher secondary	0.069	0.254
Household head's age (years)	40.749	13.037
Male household head	0.875	0.331
Household size	6.710	3.468
Rural	0.683	0.465
Household has access to piped water	0.278	0.448
Household uses flush toilet	0.283	0.450
Wealth: poorer quintile	0.213	0.409
Wealth: middle quintile	0.204	0.403
Wealth: richer quintile	0.192	0.394
Wealth: richest quintile	0.167	0.373
Percentage of households in the poorest wealth quintile at provincial level	19.867	12.552
Observations	590,488	

Mean variables estimated using de-normalized woman sampling weights are reported

Immunization of children is vital for disease prevention and control. Yet childhood immunization programs can be adversely affected by disruptions due to violent conflicts. To explore the effect of lifetime exposure to conflict on the ability to be immunized, we construct a measure of the immunization status of a child. The WHO’s Expanded Program of Immunization (EPI) recommends that all children under five receive the following basic vaccinations—BCG, Measles, DPT1, DPT2, DPT3, polio1, polio2, polio3. A child is considered to be fully immunized if they have received all of these basic vaccinations. Accordingly, we construct a dummy variable which takes 1 if a child is fully immunized, and 0 otherwise. Around 43% of children in the sample are fully immunized according to this measure (Table 2).^5^

#### Key explanatory variables

The main explanatory variables in our analysis are measures of lifetime exposure to violent conflict. As discussed earlier, these are defined based on violent events that took place within the province of a child’s residence throughout the period since their conception to the time of the interview. We exploit the information in the UCDP dataset to construct several key measures of lifetime conflict exposure.

To explore whether conflict matters for child health outcomes, we start with a binary measure of exposure, *Conflict1*. This variable takes on 1 if a child has been exposed to at least one violent event of any kind in their lifetime (*i.e.* since conception to the time observed during the interview) and 0 otherwise. Nearly 41% of children in the sample have been exposed to conflict based on this measure (Table 2).

Next, we introduce further nuance into the definition of conflict through measures that capture the length of conflict and intensity. First, we capture the length of exposure to conflict through variable *Conflict2* which represents the number of years during the period between the earliest and the latest violent events observed in a child’s province of residence throughout their lifetime. The average number of years of conflict exposure in the sample is 0.97. Second, we capture the intensity of conflict by employing a measure of the average number of deaths per year of conflict exposure, named *Conflict3*. This measure is estimated by dividing the total number of deaths that have occurred within the period between the earliest and the latest violent events observed in a child’s lifetime by the number of years in that period. Based on this conflict intensity measure, we generate a set of four dummies, distinguishing between children without exposure to a violent event that resulted in positive deaths (omitted category), and those across three different terciles of non-zero conflict-led deaths distribution (*Conflict3_cat1, Conflict3_cat2, Conflict3_cat3*).^6^

Not only does our analysis look at child health-related implications of the length and intensity of violent events, but it also asks whether the type of the conflict a child has been exposed to matters by employing two additional measures of conflict exposure. *Conflict4* is a binary variable that takes on a value of 1 if, in their lifetime, a child has been exposed to at least one violent event of Type 1 – one that features the involvement of government as one of the two parties involved in the conflict. Approximately 23% of children in the sample have had exposure to such state-based conflict in their lifetime. *Conflict5,* on the other hand, is a binary variable for lifetime exposure to one-sided violence which involves the use of armed force, be it by the government or a formally organized group, against civilians. 27% of children in the sample have been exposed to this type of conflict (Table 2).^7^

#### Control variables

Our analysis compares children with similar observable individual, household, and province characteristics. As such, we control for variables that are known to influence child stunting, underweight, and immunization status. Table 2 also provides descriptive statistics of all the variables used in the empirical analysis. These include the socio-economic and demographic profile of households (including the age and gender of the household head, household size, access to piped water and flush toilet, rural/urban residence, and wealth quintile, based on the wealth index measure available in DHS generated from data on household asset ownership using principal component analysis), an array of maternal characteristics (education levels, age, age at marriage, height, age at first birth, age at first cohabitation and current use of contraception), and information on children such as their gender, age (and its squared term), birth order, and whether they are part of multiple births.

### Empirical strategy

The aim of this paper is to empirically analyse how lifetime conflict exposure may influence child health outcomes. To identify the influence of conflict exposure variables on child health outcomes, two main models are estimated: (1) an Ordinary Least Squares (OLS) model to estimate the influence of lifetime conflict exposure on child nutrition outcomes, HAZ and WAZ; and (2) a Probit model to investigate the influence of lifetime conflict exposure on the probability of three binary outcomes: stunting, underweight and having received full immunization.^8^

In the OLS regressions, the continuous variables for child nutrition—HAZ and WAZ—are modelled as follows:

1 $$Nutrition\_cont_{icp} = \alpha Conflict_{ipc} + {\varvec{Z}}_{ipc}^{^{\prime}} {\varvec{\delta}} + {\varvec{X}}_{pc}^{^{\prime}} {\varvec{\eta}} + {\varvec{K}}_{c}^{^{\prime}} {\varvec{\zeta}} + \varepsilon_{ipc}$$

where $Nutrition\_cont_{ipc}$ is the HAZ/WAZ of child $i$ residing in province $p$ in country $c$.

Similarly, the propensity for being fully immunized, $Immunization_{ipc}^{*}$ for a child $i$ residing in province $p$ in country $c$ can be formally presented as:

2 $$Immunization_{ipc}^{*} = \beta Conflict_{ipc} + {\varvec{Z}}_{ipc}^{^{\prime}} {\varvec{\nu}} + {\varvec{X}}_{pc}^{^{\prime}} {\varvec{\gamma}} + {\varvec{K}}_{c}^{^{\prime}} {\varvec{\tau}} + \omega_{ipc}$$

Our observed outcome measure, $Immunization_{ipc}$, is assumed to relate to latent propensity through the criterion $Immunization_{ipc} = 1 \left( {Immunization_{ipc}^{*} \ge 0} \right)$ so that the probability of receiving full immunization under an assumption of normality for $\omega_{ipc}$ can be described as a probit model. To aid with interpretation, we calculate marginal effects.^9^

Child anthropometrics and immunization outcomes are modelled as a function of exposure to conflict, and an array of socio-economic, demographic, and other control variables. $Conflict_{ipc}$ is the conflict experienced by child $i$ residing in province $p$ of country $c$, from year of conception to the year observed in the DHS. Given that each child in our sample is matched with conflict at the level of sub-national provinces, we are able to include country fixed-effects in the analysis, thus child nutrition and immunization outcomes are compared within countries. In doing so, we are able to address significant sources of unobserved heterogeneity inherent to cross-country comparisons of child health outcomes. Thus, the baseline controls include country fixed-effects ${\varvec{K}}_{c}$, a vector of child/mother/household controls ${\varvec{Z}}_{ipc}$ from Table 2, and province-level controls ${\varvec{X}}_{pc}$ for the percentage of households in the poorest quintile (and percentage of households with major religion in robustness checks).

While the inclusion of province-level controls ${\varvec{X}}_{pc}$ mitigates some of the unobserved heterogeneity at the province level, it does not entirely eliminate it. Both health and conflict outcomes may be jointly driven by the same unobservables, for instance, cultural norms of provinces. Since we do observe children with varying lifetime exposure to conflict within a province, we are able to address such unobserved heterogeneity concerns by additionally controlling for province-level fixed-effects in robustness checks. We do this in the sub-sample of children who did have some exposure to conflict in their lifetime. Through this approach, we effectively identify the relationship between lifetime exposure to conflict and health outcomes exploiting the differences in exposure to violence among children living in the same province of a country.

## Results

### Descriptive statistics

In Table 3 we present the descriptive statistics of the key variables included in the analysis, disaggregated by whether or not the child has been exposed to conflict (using *Conflict1* as our variable of conflict). Approximately 41% of the children in our sample live in an area exposed to conflict.

**Table 3:** Comparison of children by conflict exposure status

	(1)	(2)	(3)	(4)	(5)
	No conflict (Conflict1 = 0)		With conflict (Conflict1 = 1)		Difference between 2 groups
	Mean	*Std. Dev*	Mean	*Std. Dev*	
HAZ	− 1.236	1.542	− 1.372	1.592	0.136***
WAZ	− 0.672	1.237	− 0.973	1.259	0.302***
Stunting (HAZ < − 2)	0.307	0.461	0.349	0.477	− 0.042***
Underweight (WAZ < − 2)	0.133	0.340	0.201	0.401	− 0.068***
Immunization (received full set of 8 recommended vaccines)	0.473	0.499	0.424	0.494	0.049***
Child's age (months)	27.840	17.198	30.941	16.963	− 3.101***
Child is male	0.505	0.500	0.505	0.500	0
Child is multiple birth	0.026	0.158	0.022	0.148	0.003***
Birth order number	2.710	1.198	2.685	1.203	0.024***
Mother's age (years)	29.133	6.796	28.964	6.707	0.169***
Mother's height (cm)	156.863	7.215	156.331	7.081	0.532***
Mother’s age at 1st birth	19.765	3.900	19.621	4.010	0.144***
Mother’s age at 1st cohabitation	19.078	4.032	18.618	4.172	0.460***
Mother is using contraception	0.431	0.495	0.407	0.491	0.023***
Mother’s education: primary	0.345	0.475	0.286	0.452	0.059***
Mother’s education: secondary	0.277	0.447	0.290	0.454	− 0.013***
Mother’s education: higher secondary	0.074	0.261	0.070	0.256	0.003***
Household head's age (years)	40.828	13.269	40.512	12.955	0.315***
Male household head	0.836	0.370	0.858	0.349	− 0.022***
Household size	6.847	3.765	6.668	3.345	0.179***
Rural	0.654	0.476	0.638	0.481	0.016***
Piped water	0.387	0.487	0.306	0.461	0.081***
Flush toilet	0.268	0.443	0.271	0.445	− 0.003*
Wealth: poorer quintile	0.229	0.420	0.214	0.410	0.014***
Wealth: middle quintile	0.209	0.406	0.190	0.392	0.019***
Wealth: richer quintile	0.175	0.380	0.179	0.384	− 0.004***
Wealth: richest quintile	0.134	0.341	0.172	0.377	− 0.037***
Observations	349,656		240,832		590,488

t-test is used to test if there is no difference between the two groups. *p* < 0.1, ** *p* < 0.05, *** *p* < 0.01

Comparing the child anthropometrics outcomes by conflict exposure in Table 3, we observe that both HAZ and WAZ are worse for children exposed to conflicts, relative to children with no exposure to conflict. In particular, the mean HAZ of a child exposed to conflict is 1.37 standard deviations below the reference, while a child with no conflict exposure is 1.24 standard deviations below the reference. Similarly, we observe statistically significant differences in WAZ across these two samples—with children exposed to conflict being 0.97 standard deviations below the reference, while children without exposure to conflict being 0.67 standard deviations below the reference. Stunting (underweight) prevalence is also 5 (7) percentage points higher for children exposed to conflict. Additionally, we observe relatively lower levels of full immunization rates among children exposed to conflict, with immunization rates being 5 percentage points lower among children who have been exposed to conflicts. Moreover, the differences in means between these groups are statistically significant across all three outcome measures.

In terms of other characteristics, Table 3 shows that there are also significant differences in maternal characteristics across these two groups. In particular, while 35% of the children in the conflict areas have mothers with no education, the figure is 30% among children with no conflict exposure. Moreover, mother’s age at first cohabitation and age at first birth is lower in the conflict-exposed sample. Turning to sanitation, access to piped water is 8 percentage points lower among children exposed to conflict, while there is hardly any difference in households’ use of flush toilet by conflict exposure status. Interestingly, 17% of the households of conflict-exposed children are in the richest quintile of wealth distribution relative to 12% for households of children who have not been exposed to conflict.

### Baseline results

We empirically analyse the relationship between child conflict exposure and health outcomes, through estimating Eqs. 1 and 2, controlling for a large set of child, mother-, household- and location-level characteristics presented in Table 2. Additionally, we include country fixed- effects.

#### Association between conflict exposure and child nutrition outcomes

In Table 4 we present OLS regression estimates on the association between children’s exposure to violent conflict events and their HAZ, across different conflict measures (for full set of estimates please see Additional file 1: Table S3). We observe a statistically significant and negative relationship between each of our measures of conflict and child HAZ scores. The coefficient of -0.043 (*p* < 0.01) for *Conflict1* indicates that at least one act of violence at the province level is associated with a child’s HAZ being 0.043 standard deviations below that of a child who was not exposed to conflict (Table 3, Column 1). Similar results are observed across different definitions of conflict (*Conflict4* and *Conflict5*). Furthermore, as we see in Table 4 (Column 2), an increase in the duration of exposure to conflict by one year (*Conflict2*) is associated with a 0.014 standard deviation decrease in HAZ score. Conflict exposure using the measure of number of deaths produces qualitatively similar results (Table 4, Column 3). Control variables such as household economic status, mother educational status, multiple births per pregnancy, birth order and child’s gender are statistically significant and have the expected signs, consistent with previous research.

**Table 4:** OLS estimates—dependent variable: HAZ

	(1)	(2)	(3)	(4)	(5)
	HAZ	HAZ	HAZ	HAZ	HAZ
Conflict1	− 0.043*** (0.007)				
Conflict2		− 0.014*** (0.002)			
Conflict3_cat1			− 0.079*** (0.009)		
Conflict3_cat2			0.000 (0.010)		
Conflict3_cat3			− 0.049*** (0.011)		
Conflict4				− 0.041*** (0.008)	
Conflict5					− 0.041*** (0.008)
Child's age	− 0.083*** (0.001)	− 0.083*** (0.001)	− 0.083*** (0.001)	− 0.083*** (0.001)	− 0.083*** (0.001)
Child's age squared	0.001*** (0.000)	0.001*** (0.000)	0.001*** (0.000)	0.001*** (0.000)	0.001*** (0.000)
Child is male	− 0.120*** (0.004)	− 0.120*** (0.004)	− 0.120*** (0.004)	− 0.120*** (0.004)	− 0.120*** (0.004)
Child is multiple birth	− 0.504*** (0.016)	− 0.504*** (0.016)	− 0.504*** (0.016)	− 0.504*** (0.016)	− 0.504*** 0.016)
Birth order number	− 0.105*** (0.003)	− 0.105*** (0.003)	− 0.106*** (0.003)	− 0.105*** (0.003)	− 0.105*** (0.003)
Mother's age	0.074*** (0.003)	0.074*** (0.003)	0.074*** (0.003)	0.074***(0.003)	0.074*** (0.003)
Mother's age squared	− 0.001*** (0.000)	− 0.001*** (0.000)	− 0.001*** (0.000)	− 0.001*** (0.000)	− 0.001*** (0.000)
Mother's height	0.045*** (0.000)	0.045*** (0.000)	0.045*** (0.000)	0.045*** (0.000)	0.045*** (0.000)
Mother’s age at 1st birth	− 0.010*** (0.001)	− 0.010*** (0.001)	− 0.010*** (0.001)	− 0.010*** (0.001)	− 0.010*** (0.001)
Mother's age at 1^st^ cohabitation	− 0.002* (0.001)	− 0.002* (0.001)	− 0.002* (0.001)	− 0.002** (0.001)	− 0.002* (0.001)
Mother is using contraception	0.057*** (0.005)	0.057*** (0.005)	0.057*** (0.005)	0.057*** (0.005)	0.057*** (0.005)
Mother’s education: primary	0.073*** (0.007)	0.072*** (0.007)	0.073*** (0.007)	0.072*** (0.006)	0.073*** (0.007)
Mother’s education: secondary education	0.217*** (0.008)	0.216*** (0.008)	0.217*** (0.008)	0.216*** (0.008)	0.217*** (0.008)
Mother’s education: higher secondary	0.334*** (0.011)	0.334*** (0.011)	0.335*** (0.011)	0.334*** (0.011)	0.335*** (0.011)
Household head's age	0.004*** (0.001)	0.004*** (0.001)	0.004*** (0.001)	0.004*** (0.001)	0.004*** (0.001)
HH head's age squared	− 0.000* (0.000)	− 0.000** (0.000)	− 0.000** (0.000)	− 0.000** (0.000)	− 0.000** (0.000)
Male household head	− 0.005 (0.005)	− 0.005 (0.005)	− 0.004 (0.005)	− 0.004 (0.005)	− 0.005 (0.005)
Household size	− 0.009*** (0.001)	− 0.009*** (0.001)	− 0.009*** (0.001)	− 0.009*** (0.001)	− 0.009*** (0.001)
Rural	− 0.063*** (0.007)	− 0.062*** (0.007)	− 0.063*** (0.007)	− 0.062*** (0.007)	− 0.063*** (0.007)
Piped water	− 0.021*** (0.006)	− 0.021*** (0.006)	− 0.020*** (0.006)	− 0.021*** (0.006)	− 0.022*** (0.006)
Flush toilet	0.126*** (0.008)	0.126*** (0.008)	0.125*** (0.008)	0.126*** (0.008)	0.126*** (0.008)
Wealth: poorer quintile	0.081*** (0.007)	0.081*** (0.007)	0.081*** (0.006)	0.082*** (0.007)	0.081*** (0.007)
Wealth: middle quintile	0.155*** (0.007)	0.155*** (0.007)	0.154*** (0.007)	0.155*** (0.007)	0.154*** (0.007)
Wealth: richer quintile	0.254*** (0.008)	0.254*** (0.008)	0.253*** (0.008)	0.254*** (0.008)	0.253*** (0.008)
Wealth: richest quintile	0.425*** (0.010)	0.425*** (0.010)	0.425*** (0.010)	0.424*** (0.010)	0.424*** (0.010)
Percentage of households in the poorest wealth quintile at provincial level	− 0.003*** (0.000)	− 0.003*** (0.000)	− 0.003*** (0.000)	− 0.003*** (0.000)	− 0.003*** (0.000)
Country dummies	Yes	Yes	Yes	Yes	Yes
Year dummies	Yes	Yes	Yes	Yes	Yes
Observations	590,488	590,488	590,488	590,488	590,488

Coefficients are reported. Standard errors in parentheses are clustered at the district level (using cluster ID). * *p* < 0.1, ** *p* < 0.05, *** *p* < 0.01

In Table 5, we present OLS estimation results from the association between exposure to violent conflict events and WAZ. As with HAZ, we observe a statistically significant and negative association between all measures of conflict exposure and WAZ among children. On average, relative to a child not exposed to conflict, a child exposed to at least one conflict event has a WAZ that is 0.071 standard deviations below the reference.

**Table 5:** OLS estimates—dependent variable: WAZ

	(1)	(2)	(3)	(4)	(5)
	WAZ	WAZ	WAZ	WAZ	WAZ
Conflict1	− 0.071*** (0.006)				
Conflict2		− 0.006*** (0.002)			
Conflict3_cat1			− 0.093*** (0.007)		
Conflict3_cat2			− 0.047*** (0.007)		
Conflict3_cat3			− 0.089*** (0.008)		
Conflict4				− 0.047*** (0.006)	
Conflict5					− 0.024*** (0.006)
Control variables	Yes	Yes	Yes	Yes	Yes
Country dummies	Yes	Yes	Yes	Yes	Yes
Year dummies	Yes	Yes	Yes	Yes	Yes
Observations	590,488	590,488	590,488	590,488	590,488

Coefficients are reported. Other control variables include those in Table 2. Standard errors in parentheses are clustered at the district level (using cluster ID). * *p* < 0.1, ** *p* < 0.05, *** *p* < 0.01

To examine the intensity of conflict on child’s WAZ score, we establish that a year’s increase in a child’s exposure to conflict is associated with 0.006 standard deviations decrease in WAZ score (Column 2). Children who have been exposed to conflict-related deaths have lower WAZ scores relative to the children not exposed to conflict (Column 3). Both state-based and one-sided violence are associated with a decrease in WAZ scores, but the magnitude of the relationship is larger in the case of a state-based conflict (there is a 0.047 reduction in WAZ score associated with exposure to at least one state-based violence).

**Fig. 3:**
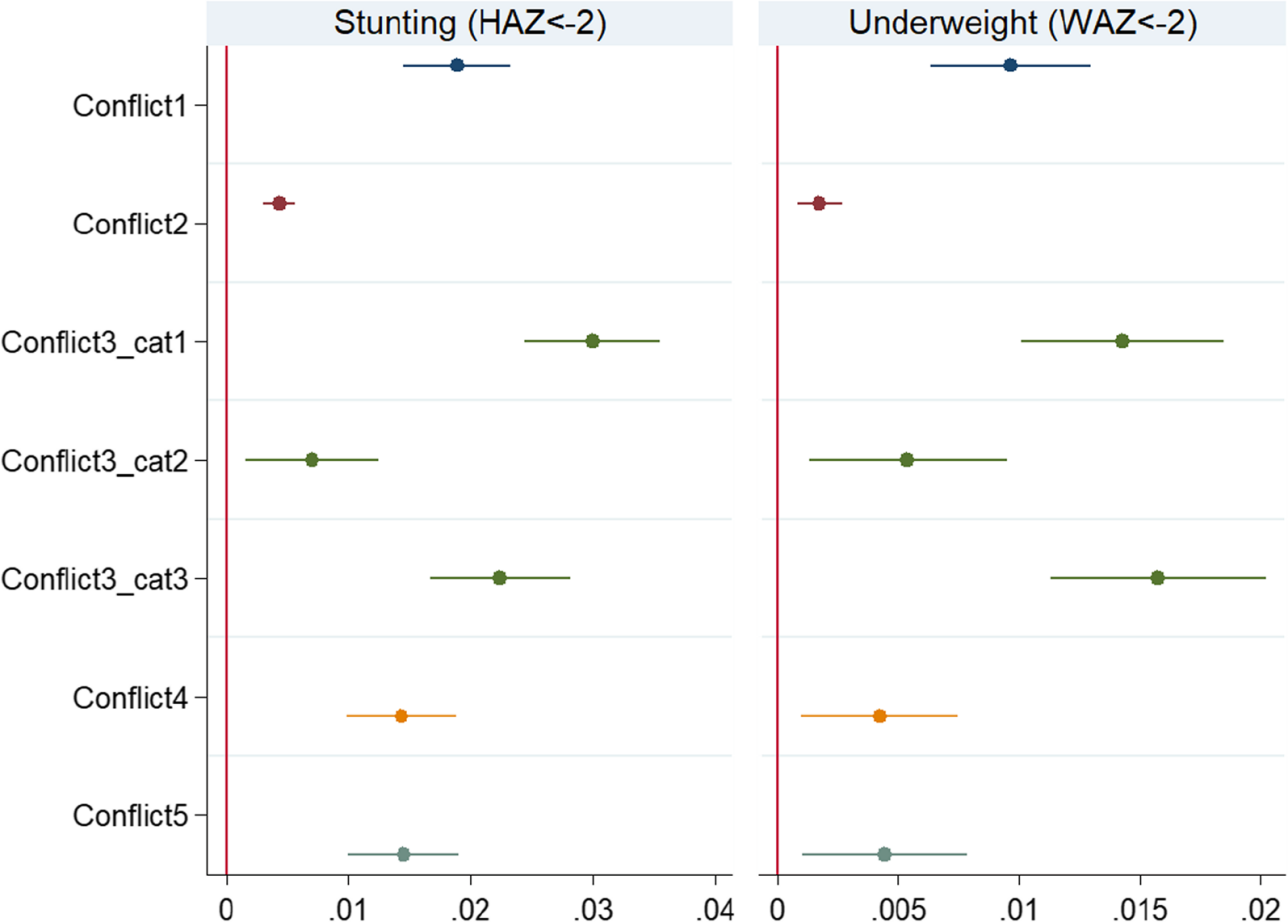
Stunting and underweight probability-marginal effects. Notes: Dependent variables: HAZ < − 2 and WAZ < − 2. Marginal effects of the conflict variables from Probit models are plotted

In Fig. 3, we graphically present the marginal effects from the Probit model for the relationship between our conflict variables and the probability of a child being stunted (HAZ < − 2) and underweight (WAZ < − 2). These marginal effects show that the probability of a child exposed to conflict being stunted is negatively signed and ranges from being between 1 percentage points (for *Conflict2*) to 3 percentage points (*Conflict3_cat1*) below that of a child not exposed to these types of conflict. The influence of conflict exposure on children’s underweight status are somewhat smaller, and range from being 0.1 percentage points (for *Conflict2*) to 1.6 percentage points (*Conflict3_cat3*) lower relative to a child not exposed to these types of conflict. Given that HAZ is a measure of long-term nutrition, the high stunting incidence among conflict exposed children is a cause for concern.

#### Association between children’s conflict exposure and childhood immunizaiton

As previously discussed, immunization of children could be adversely affected in conflict exposed areas, as health infrastructure may be negatively impacted. Our results on the association between conflict exposure and full immunization of children confirm this, with all measures of conflict being negatively signed and statistically significant (Table 6). In particular, children who have been exposed to at least one conflict event are 1.3 percentage points less likely to be fully immunized compared to those children who have not had conflict exposure in their lifetime (Column 1). The duration of conflict exposure is also important. As we see in Column 2 of Table 6, an increase in one year’s duration of conflict exposure is associated with a significant decrease in the probability of a child being fully immunized. The results for the conflict exposure defined based on the number of deaths per year (*Conflict3)* indicate that the likelihood of children missing out on receiving the recommended full immunization greatly depends on the severity of the conflict.

**Table 6:** Probit estimates—dependent variable: Immunization (received full set of 8 recommended vaccines)

	(1)	(2)	(3)	(4)	(5)
	Immunization	Immunization	Immunization	Immunization	Immunization
Conflict1	− 0.013*** (0.003)				
Conflict2		− 0.004*** (0.001)			
Conflict3_cat1			0.001 (0.003)		
Conflict3_cat2			− 0.023*** (0.003)		
Conflict3_cat3			− 0.028*** (0.004)		
Conflict4				− 0.017*** (0.003)	
Conflict5					0.005* (0.003)
Control variables	Yes	Yes	Yes	Yes	Yes
Country dummies	Yes	Yes	Yes	Yes	Yes
Year dummies	Yes	Yes	Yes	Yes	Yes
Observations	590,488	590,488	590,488	590,488	590,488

Marginal effects are reported. Other control variables include those in Table 2. Standard errors, in parentheses, are clustered at the district level (using cluster ID). * *p* < 0.1, ** *p* < 0.05, *** *p* < 0.01

### Robustness checks

Our baseline results consistently point to a negative significant relationship between child lifetime conflict exposure and their nuturitional and immunization outcomes. To test the reliability of these results, we conduct a series of robustness checks.

#### Estimates based on sample restricted to children exposed to conflicts

The influence of conflict on child health outcomes may be affected by the severity of the conflict. Therefore, we restrict the sample to only those children who live in areas where at least one violent conflict took place. From Table 7 (Panel A), we observe after imposing this sample restriction, that relative to a child who was exposed to conflicts which caused no deaths, children exposed to *Conflict3_cat 1* to *Conflict3_cat 3* had poorer nutritional outcomes, measured using child anthropometrics. However, within this sample there is no statistically significant difference in immunization outcomes by conflict exposure intensity measures.

**Table 7:** Robustness test: Sample limited to children exposed to conflict (conflict1 = 1)

	(1)	(2)	(3)	(4)	(5)
	HAZ [Coefficient]	WAZ [Coefficient]	Stunting (HAZ < − 2) [Marginal effect]	Underweight (HAZ < − 2) [Marginal effect]	Immunization [Marginal effect]
*Reference category: Children exposed to conflict with no death (Conflict3 = 0)*					
Panel A: Without province fixed effects					
Conflict3_cat1	− 0.084*** (0.022)	− 0.090*** (0.016)	0.037*** (0.007)	0.023*** (0.005)	0.013 (0.008)
Conflict3_cat2	− 0.014 (0.022)	− 0.051*** (0.016)	0.017** (0.007)	0.013** (0.005)	− 0.002 (0.008)
Conflict3_cat3	− 0.043* (0.023)	− 0.077*** (0.017)	0.029*** (0.007)	0.019*** (0.005)	0.005 (0.008)
Control variables	Yes	Yes	Yes	Yes	Yes
Country dummies	Yes	Yes	Yes	Yes	Yes
Year dummies	Yes	Yes	Yes	Yes	Yes
Province dummies	No	No	No	No	No
Panel B: With province fixed effects					
Conflict3_cat1	− 0.016 (0.031)	− 0.058** (0.025)	0.039*** (0.009)	0.022*** (0.008)	− 0.006 (0.011)
Conflict3_cat2	0.021 (0.031)	− 0.066*** (0.025)	0.024*** (0.009)	0.020** (0.008)	− 0.026** (0.011)
Conflict3_cat3	− 0.058* (0.033)	− 0.124*** (0.027)	0.043*** (0.010)	0.029*** (0.009)	− 0.001 (0.011)
Control variables	Yes	Yes	Yes	Yes	Yes
Country dummies	Yes	Yes	Yes	Yes	Yes
Year dummies	Yes	Yes	Yes	Yes	Yes
Province dummies	Yes	Yes	Yes	Yes	Yes
Observations	240,832	240,832	240,832	240,832	240,832

OLS coefficients are reported in columns 1–2 and probit marginal effects in columns 3–5. Control variables include those in Table 2. Standard errors, in parentheses, are clustered at the district level (using cluster ID). * *p* < 0.1, ** *p* < 0.05, *** *p* < 0.01

#### Estimates addressing unobserved heterogeneity

Our baseline regression controls for a large set of characteristics of individuals, their mothers, households and locations. In particular, we include a control variable that measures the percentage of households in the poorest quintile at the province level. In an extended analysis (Additional file 1: Table S4), we also generated an additional measure of province characteristics: the percentage of households with major religion^10^ (while also controlling for individual households’ own religion status). Inclusion of such province-level religion measure potentially controls for unobserved cultural charactierstics that could be correlated with both conflict exposure and health outcomes. This information is not always available, thus the sample was restricted based on availabaility of religion information. The results were largely robust to augmenting the model with this additional province-level variable, in spite of the smaller sample size (Additional file 1: Table S4). The rationale behind inclusion of these additional province-level control variables is to mitigate some of the unobserved heterogeneity at the province level, given that both heath and conflict outcomes may be jointly driven by the same unobservables.

To address the issue of such unobserved heterogeneity more convincingly, in Panel B of Table 7 we re-estimate the regressions from Panel A while additionally controlling for province fixed-effects. What this approach effectively achieves is to compare children within the same province of the same country who vary in the intensity of their conflict exposure.

Furthermore, after controlling for province fixed-effects, the results indicate that, compared to a child who was exposed to conflict which caused no deaths, children exposed to *Conflict3_cat1* to *Conflict3_cat 3* had poorer nutritional outcomes. The direction of results is also similar in the case of children's full immunization.

#### Estimates based on geo-coded data

The heterogeneity in province size across countries may potentially have implications for the results. As shown in Additional file 1: Fig. S1, there are significant differences in average province size by country, as proxied by province population size. To account for this heterogeneity to an extent, we augment the regressions with a measure of province population size – see Additional file 1: Table S5 which shows that the results are not sensitive to this change in model specification.

To better identify conflict exposure, we conduct additional analyses including geocoded data for the sample of children who were exposed to conflict from the year of conception to the survey year. We note that geocoded data are only available for 238,851 children. We calculate the distance in thousands of kilometers from each child’s residence to the nearest conflict occurence from conception year to survey year, and use a log term of the distance in the analyses. The results presented in Table 8 are keeping in line with our main results.

**Table 8:** Results using geocoded data including only children exposed to conflict

	(1)	(2)	(3)	(4)	(5)
	HAZ [OLS]	WAZ [OLS]	Stunting [Probit]	Underweight [Probit]	Immunization [Probit]
*Panel A: Include country dummies*					
Ln of distance (000 km) to the nearest conflict	− 0.004 (0.003)	− 0.006** (0.003)	− 0.001 (0.001)	− 0.000 (0.001)	− 0.006*** (0.001)
Control variables	Yes	Yes	Yes	Yes	Yes
Country dummies	Yes	Yes	Yes	Yes	Yes
Adjusted *R*^2^	0.18	0.17			
*Panel B: Include province dummies*					
Ln of distance (000 km) to the nearest conflict	− 0.012*** (0.004)	− 0.004 (0.003)	0.002** (0.001)	− 0.001 (0.001)	− 0.005*** (0.001)
Control variables	Yes	Yes	Yes	Yes	Yes
Province dummies	Yes	Yes	Yes	Yes	Yes
Adjusted *R*^2^	0.20	0.18			
Observations	238,851	238,851	238,851	238,851	238,851

Other control variables include those in Table 2. Coefficients are reported in columns 1 and 2, marginal effects are reported in columns 3–5. Standard errors in parentheses are clustered at the district level (using cluster ID). * *p* < 0.1, ** *p* < 0.05, *** *p* < 0.01

#### Estimates based on the sample with no migration history

Our measures of conflict exposure are linked to a child’s location of residence at the time of the interview, and we derive their exposure retrospectively based on the timing of their conception. As such, we assume an absence of migration, which may be a strong assumption for some settings. To mitigate the potential problems associated with such assumption, as a final robustness check, we limit the analysis sample to children with no migration history. As the results reported in Table 9 show, the negative significant relationship between conflict exposure measures and child health outcomes largely persists within the non-migrant sample.

**Table 9:** Robustness test: Sample limited to children from non-migrant households

		Explanatory conflict variable						
Dependent variable		(1)	(2)	(3)	(4)	(5)	(6)	(7)
		Conflict1	Conflict2	Conflict3_cat1	Conflict3_cat2	Conflict3_cat3	Conflict4	Conflict5
HAZ	Coefficient	− 0.063*** (0.010)	− 0.007*** (0.003)	− 0.075*** (0.013)	− 0.062*** (0.012)	− 0.043*** (0.014)	− 0.033*** (0.010)	− 0.022* (0.011)
WAZ	Coefficient	− 0.083*** (0.008)	− 0.003* (0.002)	− 0.094*** (0.010)	− 0.091*** (0.010)	− 0.061*** (0.011)	− 0.042*** (0.008)	− 0.011 (0.009)
Stunting (HAZ<− 2)	Marginal effect	0.023*** (0.003)	0.004*** (0.001)	0.029*** (0.004)	0.019*** (0.004)	0.020*** (0.004)	0.012*** (0.003)	0.011*** (0.003)
Underweight (WAZ<− 2)	Marginal effect	0.009*** (0.002)	0.000 (0.001)	0.006** (0.003)	0.013*** (0.003)	0.008*** (0.003)	− 0.002 (0.002)	− 0.001 (0.002)
Immunization	Marginal effect	0.006* (0.004)	0.001 (0.001)	0.033*** (0.004)	− 0.024*** (0.005)	0.004 (0.005)	− 0.004 (0.004)	0.005 (0.004)
Control variables		Yes	Yes	Yes	Yes	Yes	Yes	Yes
Country dummies		Yes	Yes	Yes	Yes	Yes	Yes	Yes
Year dummies		Yes	Yes	Yes	Yes	Yes	Yes	Yes
Observations		315100	315100	315100	315100	315100	315100	315100

OLS coefficients and probit marginal effects are reported. Columns (3)–(5) refer to coefficients/marginal effects from the same regression where Conflict3_cat1-Conflict3_cat3 are jointly included as controls. Other control variables include those in Table 2. Standard errors, in parentheses, are clustered at the district level (using cluster ID). * *p* < 0.1, ** *p* < 0.05, *** *p* < 0.01

Additionally, we conducted a series of robustness exercises where we estimated our models disaggregated by world regions, rural/urban residence and wealth quintiles. We also established the robustness of our results to inclusion of India in the sample. These results are not reported here due to space considerations but are presented in the Additional file 1: Tables S7–S9.

## Conclusions

In this paper, we use geo-referenced data on three types of violent conflicts (*i.e. state-based, non-state, and one-sided* conflicts) from the *Uppsala Conflict Data Program (UCDP)* Georeferenced Event Dataset, to link the location of conflict incidents (both levels and severity of conflicts) with mother-children pairs using nationally representative household-level data from the *Demographic Health Surveys (DHS),* which use a uniform questionnaire across all countries and over time. This allows us to construct a large unique database involving children from 52 countries over the period 1997–2018 to empirically test if exposure to violent conflict events has adverse outcomes on children’s nutrition outcomes (using HAZ and WAZ), and access to immunization services.

Our study contributes to the existing literature, which has largely focused on single country-studies, used a single measure of conflict, or a single outcome measure. To the best of our knowledge, ours is the first large-scale multi-country study on the role of conflict on child nutrition and immunization outcomes, over a twenty year period. Our findings are in keeping with previous research, and are robust across a range of specifications, alternative measures of conflict and sub-samples. Moreover, the inclusion of a large range of countries over a long period of time, allows us to highlight key common factors that mediate the links between conflict exposure and child nutrition and immunization outcomes.

We find that there are statistically significant differences in all three child health measures between children exposed to conflict and those living in non-conflict areas, with children living in conflict areas having worse health outcomes. Our empirical evidence shows that even after controlling for a large array of socio-economic and demographic characteristics, conflict exposure is negatively associated with child nutrition and immunization, across all our measures of conflict.

Our results suggest that child health needs to be an area of significant focus for policy makers in conflict-exposed areas. In particular, given that HAZ is a measure of long-term nutrition, the high stunting incidence among conflict exposed children shows that there is an urgent need to strengthen health systems in conflict exposed areas. Our discussion of mechanisms potentially mediating the link between conflict and child health suggests that the destruction of health systems, infrastructure, and disruption to services can potentially play a role, and thus should be prioritised in policy interventions. In particular, policy makers must ensure that even during conflict periods, some maternal and child health services should be maintained. Additional areas of policy intervention that may help in mitigating some of the adverse effects of conflict on child health include improvements in access to health care for both children as well as their carers, female education, and household living conditions, access to information on nutritious food.

Our study also suffers from some limitations. The main one is that this is a large multi-country study, and it is likely that we were not able to fully capture some country-specifc issues. Furthermore, the vaccination data does not allow us to identify the precise date of the vaccination.

## Supplementary Information

**Additional file 1: Table S1.** Sample characteristics by country. Table S2: Conflict status during 1997 to 2018 by country. Table S3: OLS estimates, full set of results - dependent variable: HAZ. Table S4: Robustness test: Controlling for religion. Table S5: Robustness test: controlling for a proxy of provincial population. Table S6: Results including India. Table S7: Results disaggregated by world regions. Table S8: Results disaggregated by rural/urban residence. Table S9: Results disaggregated by household wealth quintiles. Table S10: The association between conflict intensity and child health outcomes. Fig. S1. Mean of provinces’ estimated population by country

## Data Availability

The datasets used in the analysis are publicly available upon registration.

## Footnotes

1 Available from: https://dhsprogram.com/what-we-do/survey-Types/dHs.cfm. The 16 countries with DHS surveys conducted before 2003 include Brazil, Central African, Ecuador, El Salvador, Kazakhstan, Mexico, Nicaragua, Nigeria Ondo, Paraguay, Sri Lanka, Sudan, Thailand, Trinidad, Tunisia, Uzbekistan, Vietnam.

2 However, we did estimate models including India, and these results are presented in Table S6 of the Supplementary appendix. Given the large size of the country and the fact that the provinces are actually entire states, we have dropped zero conflict-exposed individuals and estimated the association between conflict and child nutrition outcomes.

3 To safeguard respondent’s confidentiality, DHS randomly displays the GPS latitude/longitude positions, up to 2 km in urban areas and 5 km in rural areas.

4 The reference groups are identified by the 2006 World Health Organization (WHO)’s Growth Standards which include children who are at the same age and sex and raised in a seemingly optimal environment, controlling for discrepancies in other socio-economic characteristics.

5 We have matched the year of conception of the child with the year of conflict. Given that the information on the date of children's immunization is available for only the last birth, the information is missing for most of the children. Moreover, our outcome variable is full immunization of children, does not capture the timing of the vaccination.

6 In robustness checks, following previous research [29], we additionally employ a continuous measure of conflict intensity defined as the square root of deaths per capita. The results are in line with our main results and are presented in Table S10.

7 While UCDP also contains information on Type 2 non-state conflict, it makes up only a very small share (around 10%) of the total number of violence events taking place within our sample and is therefore not considered in this analysis.

8 To account for potential spatial dependence, we cluster the standard errors at the level of districts.

9 Models of similar form are estimated for the the propensity for stunting/underweight, ${Nutrition\_binary}_{ipc}^{*}$.

10 Major religion is defined as the religion followed by the largest share of surveyed housesholds in each province.
