# Supplementary material for: Exposure to conflict and child health outcomes: evidence from a large multi-country study
Source: Confl Health. 2022 Oct 10;16:52. doi: 10.1186/s13031-022-00483-9 (PMC9845514; doi:10.1186/s13031-022-00483-9)

**Supplementary tables**

Table S1: Sample characteristics by country

| No. | Country | No. of observations | % of observations in total sample | Mean HAZ | Mean WAZ | Stunting incidence (HAZ<-2) | Underweight incidence (WAZ<-2) | Full immunization incidence |
| --- | --- | --- | --- | --- | --- | --- | --- | --- |
| 1 | Albania | 3,658 | 0.62 | -0.429 | 0.283 | 0.151 | 0.030 | 0.291 |
| 2 | Armenia | 2,737 | 0.46 | -0.401 | 0.161 | 0.120 | 0.029 | 0.484 |
| 3 | Azerbaijan | 1,906 | 0.32 | -1.066 | -0.423 | 0.262 | 0.077 | 0.413 |
| 4 | Bangladesh | 25,496 | 4.32 | -1.657 | -1.597 | 0.411 | 0.365 | 0.703 |
| 5 | Bolivia | 8,527 | 1.44 | -1.397 | -0.332 | 0.334 | 0.050 | 0.552 |
| 6 | Burkina Faso | 14,024 | 2.37 | -1.431 | -1.332 | 0.381 | 0.291 | 0.498 |
| 7 | Burundi | 9,079 | 1.54 | -2.066 | -1.374 | 0.543 | 0.275 | 0.488 |
| 8 | Cameroon | 7,673 | 1.30 | -1.268 | -0.604 | 0.331 | 0.130 | 0.445 |
| 9 | Chad | 13,703 | 2.32 | -1.451 | -1.289 | 0.407 | 0.304 | 0.166 |
| 10 | Colombia | 24,943 | 4.22 | -0.881 | -0.320 | 0.155 | 0.046 | 0.559 |
| 11 | Comoros | 2,310 | 0.39 | -0.989 | -0.672 | 0.269 | 0.131 | 0.445 |
| 12 | Congo | 7,526 | 1.27 | -1.069 | -0.668 | 0.268 | 0.113 | 0.376 |
| 13 | DR Congo | 10,481 | 1.77 | -1.549 | -1.049 | 0.430 | 0.219 | 0.301 |
| 14 | Egypt | 34,984 | 5.92 | -0.776 | -0.166 | 0.237 | 0.056 | 0.609 |
| 15 | Eswatini | 1,332 | 0.23 | -1.157 | -0.226 | 0.265 | 0.042 | 0.697 |
| 16 | Ethiopia | 17,891 | 3.03 | -1.415 | -1.240 | 0.383 | 0.264 | 0.211 |
| 17 | Gambia | 2,942 | 0.50 | -1.063 | -1.029 | 0.251 | 0.175 | 0.608 |
| 18 | Ghana | 7,711 | 1.31 | -1.118 | -0.819 | 0.271 | 0.141 | 0.579 |
| 19 | Guatemala | 10,316 | 1.75 | -1.832 | -0.795 | 0.454 | 0.117 | 0.663 |
| 20 | Guinea | 8,637 | 1.46 | -1.151 | -0.857 | 0.321 | 0.173 | 0.226 |
| 21 | Guyana | 1,413 | 0.24 | -1.035 | -0.531 | 0.229 | 0.094 | 0.521 |
| 22 | Haiti | 9,752 | 1.65 | -1.016 | -0.652 | 0.231 | 0.116 | 0.314 |
| 23 | Honduras | 18,144 | 3.07 | -1.343 | -0.544 | 0.301 | 0.088 | 0.717 |
| 24 | Jordan | 14,965 | 2.53 | -0.492 | -0.090 | 0.109 | 0.037 | 0.531 |
| 25 | Kenya | 17,425 | 2.95 | -1.232 | -0.775 | 0.305 | 0.145 | 0.530 |
| 26 | Kyrgyzstan | 3,987 | 0.68 | -0.823 | -0.121 | 0.180 | 0.035 | 0.657 |
| 27 | Lesotho | 3,749 | 0.63 | -1.568 | -0.750 | 0.387 | 0.137 | 0.553 |
| 28 | Liberia | 6,440 | 1.09 | -1.341 | -0.844 | 0.344 | 0.154 | 0.302 |
| 29 | Madagascar | 4,122 | 0.70 | -1.794 | -1.423 | 0.479 | 0.318 | 0.495 |
| 30 | Mali | 18,747 | 3.17 | -1.271 | -1.119 | 0.344 | 0.235 | 0.315 |
| 31 | Moldova | 1,276 | 0.22 | -0.257 | 0.051 | 0.113 | 0.034 | 0.736 |
| 32 | Morocco | 5,328 | 0.90 | -0.819 | -0.304 | 0.225 | 0.092 | 0.756 |
| 33 | Mozambique | 16,081 | 2.72 | -1.659 | -0.839 | 0.419 | 0.148 | 0.539 |
| 34 | Myanmar | 4,155 | 0.70 | -1.335 | -1.064 | 0.304 | 0.174 | 0.510 |
| 35 | Namibia | 3,282 | 0.56 | -1.157 | -0.832 | 0.278 | 0.154 | 0.590 |
| 36 | Nepal | 9,823 | 1.66 | -1.749 | -1.532 | 0.445 | 0.332 | 0.621 |
| 37 | Niger | 8,168 | 1.38 | -1.661 | -1.474 | 0.433 | 0.338 | 0.353 |
| 38 | Nigeria | 55,710 | 9.43 | -1.332 | -1.052 | 0.366 | 0.230 | 0.192 |
| 39 | Pakistan | 6,907 | 1.17 | -1.514 | -1.080 | 0.390 | 0.219 | 0.357 |
| 40 | Peru | 53,575 | 9.07 | -1.285 | -0.339 | 0.266 | 0.050 | 0.470 |
| 41 | Rwanda | 10,437 | 1.77 | -1.719 | -0.741 | 0.436 | 0.121 | 0.578 |
| 42 | Senegal | 6,213 | 1.05 | -1.083 | -0.956 | 0.261 | 0.171 | 0.489 |
| 43 | Sierra Leone | 5,418 | 0.92 | -1.240 | -0.730 | 0.356 | 0.157 | 0.508 |
| 44 | South Africa | 445 | 0.08 | -1.032 | -0.195 | 0.234 | 0.045 | 0.303 |
| 45 | Tajikistan | 10,238 | 1.73 | -0.876 | -0.604 | 0.203 | 0.093 | 0.495 |
| 46 | Tanzania | 21,725 | 3.68 | -1.550 | -0.922 | 0.379 | 0.150 | 0.428 |
| 47 | Togo | 3,062 | 0.52 | -1.246 | -0.948 | 0.277 | 0.167 | 0.503 |
| 48 | Turkey | 5,047 | 0.62 | -0.570 | 0.130 | 0.167 | 0.040 | 0.354 |
| 49 | Uganda | 8,441 | 0.46 | -1.272 | -0.710 | 0.310 | 0.122 | 0.260 |
| 50 | Yemen | 13,180 | 0.32 | -1.725 | -1.581 | 0.443 | 0.360 | 0.338 |
| 51 | Zambia | 15,008 | 4.32 | -1.557 | -0.847 | 0.407 | 0.140 | 0.490 |
| 52 | Zimbabwe | 12,349 | 1.44 | -1.276 | -0.608 | 0.294 | 0.095 | 0.444 |

Table S2: Conflict status during 1997 to 2018 by country

| No. | Country | No. of all conflicts | No. of type 1 conflict | No. of type 2 conflict | No. of type 3 conflict | Total number of deaths | Start year of the earliest conflict | End year of the latest conflict |
| --- | --- | --- | --- | --- | --- | --- | --- | --- |
| 1 | Albania | 4 | 4 | 0 | 0 | 6 | 1999 | 1999 |
| 2 | Armenia | 19 | 18 | 0 | 1 | 34 | 2005 | 2018 |
| 3 | Azerbaijan | 283 | 283 | 0 | 0 | 552 | 1997 | 2018 |
| 4 | Bangladesh | 429 | 215 | 158 | 56 | 720 | 1997 | 2018 |
| 5 | Bolivia | 10 | 0 | 10 | 0 | 52 | 1998 | 2000 |
| 6 | Burkina Faso | 68 | 13 | 0 | 55 | 206 | 2016 | 2018 |
| 7 | Burundi | 1,296 | 748 | 24 | 524 | 12,126 | 1997 | 2018 |
| 8 | Cameroon | 599 | 313 | 22 | 264 | 4,254 | 1998 | 2018 |
| 9 | Chad | 245 | 129 | 19 | 97 | 6,923 | 1997 | 2018 |
| 10 | Colombia | 3,904 | 2,903 | 157 | 844 | 20,292 | 1997 | 2018 |
| 11 | Comoros | 5 | 2 | 3 | 0 | 96 | 1997 | 1998 |
| 12 | Congo | 211 | 119 | 0 | 92 | 15,525 | 1997 | 2018 |
| 13 | DR Congo | 3,515 | 1,092 | 448 | 1,975 | 73,275 | 1997 | 2018 |
| 14 | Egypt | 454 | 333 | 74 | 47 | 3,849 | 1997 | 2018 |
| 15 | Eswatini | 0 | 0 | 0 | 0 | 0 |  |  |
| 16 | Ethiopia | 1,714 | 1,119 | 255 | 340 | 87,882 | 1997 | 2018 |
| 17 | Gambia | 2 | 0 | 0 | 2 | 8 | 2005 | 2005 |
| 18 | Ghana | 16 | 0 | 16 | 0 | 180 | 2000 | 2010 |
| 19 | Guatemala | 25 | 0 | 14 | 11 | 163 | 1997 | 2015 |
| 20 | Guinea | 78 | 24 | 5 | 49 | 1,073 | 1998 | 2018 |
| 21 | Guyana | 3 | 0 | 0 | 3 | 29 | 2008 | 2008 |
| 22 | Haiti | 32 | 19 | 0 | 13 | 304 | 1999 | 2018 |
| 23 | Honduras | 32 | 0 | 27 | 5 | 236 | 1999 | 2016 |
| 24 | Jordan | 5 | 4 | 0 | 1 | 93 | 2005 | 2016 |
| 25 | Kenya | 840 | 141 | 450 | 249 | 4,851 | 1997 | 2018 |
| 26 | Kyrgyzstan | 47 | 22 | 25 | 0 | 238 | 1999 | 2010 |
| 27 | Lesotho | 4 | 4 | 0 | 0 | 68 | 1998 | 1998 |
| 28 | Liberia | 136 | 74 | 0 | 62 | 6,218 | 1997 | 2003 |
| 29 | Madagascar | 43 | 0 | 38 | 5 | 208 | 2002 | 2017 |
| 30 | Mali | 637 | 425 | 101 | 111 | 4,238 | 1997 | 2018 |
| 31 | Moldova | 0 | 0 | 0 | 0 | 0 |  |  |
| 32 | Morocco | 2 | 0 | 0 | 2 | 34 | 2003 | 2003 |
| 33 | Mozambique | 77 | 39 | 0 | 38 | 272 | 2004 | 2018 |
| 34 | Myanmar | 2,202 | 924 | 58 | 1,220 | 11,649 | 1997 | 2018 |
| 35 | Namibia | 18 | 8 | 0 | 10 | 72 | 1999 | 2002 |
| 36 | Nepal | 5,585 | 3,669 | 4 | 1,912 | 12,259 | 1997 | 2009 |
| 37 | Niger | 155 | 102 | 9 | 44 | 1,684 | 1997 | 2018 |
| 38 | Nigeria | 4,026 | 1,436 | 1,353 | 1,237 | 51,798 | 1997 | 2018 |
| 39 | Pakistan | 5,595 | 4,702 | 360 | 533 | 40,568 | 1997 | 2018 |
| 40 | Peru | 143 | 117 | 0 | 26 | 518 | 1997 | 2017 |
| 41 | Rwanda | 204 | 96 | 0 | 108 | 8,591 | 1997 | 2018 |
| 42 | Senegal | 186 | 120 | 4 | 62 | 1,220 | 1997 | 2018 |
| 43 | Sierra Leone | 775 | 361 | 0 | 414 | 11,811 | 1997 | 2002 |
| 44 | South Africa | 65 | 0 | 61 | 4 | 99 | 1997 | 2016 |
| 45 | Tajikistan | 75 | 74 | 0 | 1 | 906 | 1997 | 2018 |
| 46 | Tanzania | 12 | 0 | 1 | 11 | 52 | 1997 | 2018 |
| 47 | Togo | 97 | 0 | 0 | 97 | 445 | 1998 | 2005 |
| 48 | Turkey | 2,526 | 2,453 | 1 | 72 | 12,450 | 1997 | 2018 |
| 49 | Uganda | 1,427 | 884 | 96 | 447 | 11,607 | 1997 | 2018 |
| 50 | Yemen | 2,225 | 1,854 | 326 | 45 | 24,767 | 2003 | 2018 |
| 51 | Zambia | 3 | 0 | 0 | 3 | 8 | 2000 | 2001 |
| 52 | Zimbabwe | 51 | 0 | 0 | 51 | 261 | 1998 | 2018 |
|  | Total | 40,105 | 24,843 | 4,119 | 11,143 | 434,770 |  |  |

Table S3: OLS estimates, full set of results - dependent variable: HAZ

|  | (1) | (2) | (3) | (4) | (5) |
| --- | --- | --- | --- | --- | --- |
|  | HAZ | HAZ | HAZ | HAZ | HAZ |
| Conflict1 | -0.043*** (0.007) |  |  |  |  |
| Conflict2 |  | -0.014*** (0.002) |  |  |  |
| Conflict3_cat1 |  |  | -0.079*** (0.009) |  |  |
| Conflict3_cat2 |  |  | 0.000 (0.010) |  |  |
| Conflict3_cat3 |  |  | -0.049*** (0.011) |  |  |
| Conflict4 |  |  |  | -0.041*** (0.008) |  |
| Conflict5 |  |  |  |  | -0.041*** (0.008) |
| Child's age (months) | -0.083*** (0.001) | -0.083*** (0.001) | -0.083*** (0.001) | -0.083*** (0.001) | -0.083*** (0.001) |
| Child's age squared | 0.001*** (0.000) | 0.001*** (0.000) | 0.001*** (0.000) | 0.001*** (0.000) | 0.001*** (0.000) |
| Child is male | -0.120*** (0.004) | -0.120*** (0.004) | -0.120*** (0.004) | -0.120*** (0.004) | -0.120*** (0.004) |
| Child is multiple birth | -0.504*** (0.016) | -0.504*** (0.016) | -0.504*** (0.016) | -0.504*** (0.016) | -0.504*** (0.016) |
| Birth order number | -0.105*** (0.003) | -0.105*** (0.003) | -0.106*** (0.003) | -0.105*** (0.003) | -0.105*** (0.003) |
| Mother's age (years) | 0.074*** (0.003) | 0.074*** (0.003) | 0.074*** (0.003) | 0.074*** (0.003) | 0.074*** (0.003) |
| Mother's age squared | -0.001*** (0.000) | -0.001*** (0.000) | -0.001*** (0.000) | -0.001*** (0.000) | -0.001*** (0.000) |
| Mother's height (cm) | 0.045*** (0.000) | 0.045*** (0.000) | 0.045*** (0.000) | 0.045*** (0.000) | 0.045*** (0.000) |
| Mother’s age at 1st birth | -0.010*** (0.001) | -0.010*** (0.001) | -0.010*** (0.001) | -0.010*** (0.001) | -0.010*** (0.001) |
| Mother's age at first cohabitation | -0.002* (0.001) | -0.002* (0.001) | -0.002* (0.001) | -0.002** (0.001) | -0.002* (0.001) |
| Mother is using contraception | 0.057*** (0.005) | 0.057*** (0.005) | 0.057*** (0.005) | 0.057*** (0.005) | 0.057*** (0.005) |
| Mother completed primary education | 0.073*** (0.007) | 0.072*** (0.007) | 0.073*** (0.007) | 0.072*** (0.006) | 0.073*** (0.007) |
| Mother completed secondary education | 0.217*** (0.008) | 0.216*** (0.008) | 0.217*** (0.008) | 0.216*** (0.008) | 0.217*** (0.008) |
| Mother completed higher secondary education | 0.334*** (0.011) | 0.334*** (0.011) | 0.335*** (0.011) | 0.334*** (0.011) | 0.335*** (0.011) |
| Household head's age (years) | 0.004*** (0.001) | 0.004*** (0.001) | 0.004*** (0.001) | 0.004*** (0.001) | 0.004*** (0.001) |
| HH head's age squared | -0.000* (0.000) | -0.000** (0.000) | -0.000** (0.000) | -0.000** (0.000) | -0.000** (0.000) |
| Male household head | -0.005 (0.005) | -0.005 (0.005) | -0.004 (0.005) | -0.004 (0.005) | -0.005 (0.005) |
| Household size | -0.009*** (0.001) | -0.009*** (0.001) | -0.009*** (0.001) | -0.009*** (0.001) | -0.009*** (0.001) |
| Rural | -0.063*** (0.007) | -0.062*** (0.007) | -0.063*** (0.007) | -0.062*** (0.007) | -0.063*** (0.007) |
| Household access piped water | -0.021*** (0.006) | -0.021*** (0.006) | -0.020*** (0.006) | -0.021*** (0.006) | -0.022*** (0.006) |
| Household use flush toilet | 0.126*** (0.008) | 0.126*** (0.008) | 0.125*** (0.008) | 0.126*** (0.008) | 0.126*** (0.008) |
| Wealth: poorer quintile | 0.081*** (0.007) | 0.081*** (0.007) | 0.081*** (0.006) | 0.082*** (0.007) | 0.081*** (0.007) |
| Wealth: middle quintile | 0.155*** (0.007) | 0.155*** (0.007) | 0.154*** (0.007) | 0.155*** (0.007) | 0.154*** (0.007) |
| Wealth: richer quintile | 0.254*** (0.008) | 0.254*** (0.008) | 0.253*** (0.008) | 0.254*** (0.008) | 0.253*** (0.008) |
| Wealth: richest quintile | 0.425*** (0.010) | 0.425*** (0.010) | 0.425*** (0.010) | 0.424*** (0.010) | 0.424*** (0.010) |
| Percentage of households in the poorest wealth quintile at provincial level | -0.003*** (0.000) | -0.003*** (0.000) | -0.003*** (0.000) | -0.003*** (0.000) | -0.003*** (0.000) |
| Armenia | 0.005 (0.050) | 0.002 (0.050) | 0.008 (0.050) | -0.001 (0.050) | 0.004 (0.050) |
| Azerbaijan | -0.377*** (0.063) | -0.386*** (0.063) | -0.366*** (0.063) | -0.381*** (0.063) | -0.392*** (0.063) |
| Bangladesh | -0.625*** (0.038) | -0.630*** (0.037) | -0.595*** (0.038) | -0.650*** (0.037) | -0.636*** (0.037) |
| Bolivia | -0.202*** (0.040) | -0.197*** (0.040) | -0.201*** (0.040) | -0.205*** (0.040) | -0.194*** (0.040) |
| Burkina Faso | -0.642*** (0.040) | -0.633*** (0.040) | -0.636*** (0.040) | -0.642*** (0.040) | -0.631*** (0.040) |
| Burundi | -1.202*** (0.037) | -1.212*** (0.036) | -1.195*** (0.037) | -1.213*** (0.036) | -1.202*** (0.037) |
| Cameroon | -0.552*** (0.041) | -0.548*** (0.041) | -0.551*** (0.041) | -0.555*** (0.041) | -0.545*** (0.041) |
| Chad | -0.817*** (0.041) | -0.817*** (0.041) | -0.814*** (0.041) | -0.822*** (0.041) | -0.809*** (0.041) |
| Colombia | -0.036 (0.036) | -0.027 (0.036) | -0.032 (0.036) | -0.039 (0.036) | -0.035 (0.036) |
| Comoros | -0.154*** (0.054) | -0.148*** (0.054) | -0.155*** (0.054) | -0.154*** (0.054) | -0.147*** (0.054) |
| Congo | -0.263*** (0.043) | -0.267*** (0.042) | -0.267*** (0.043) | -0.266*** (0.042) | -0.262*** (0.043) |
| DR Congo | -0.718*** (0.043) | -0.726*** (0.043) | -0.713*** (0.043) | -0.743*** (0.043) | -0.713*** (0.044) |
| Egypt | -0.241*** (0.039) | -0.247*** (0.039) | -0.238*** (0.039) | -0.252*** (0.039) | -0.244*** (0.039) |
| Eswatini | -0.377*** (0.051) | -0.377*** (0.051) | -0.377*** (0.051) | -0.379*** (0.051) | -0.374*** (0.051) |
| Ethiopia | -0.393*** (0.040) | -0.392*** (0.040) | -0.395*** (0.040) | -0.408*** (0.040) | -0.390*** (0.041) |
| Gambia | -0.422*** (0.048) | -0.422*** (0.048) | -0.419*** (0.048) | -0.435*** (0.048) | -0.413*** (0.048) |
| Ghana | -0.354*** (0.040) | -0.350*** (0.040) | -0.350*** (0.040) | -0.357*** (0.040) | -0.346*** (0.040) |
| Guatemala | -0.816*** (0.042) | -0.816*** (0.041) | -0.811*** (0.042) | -0.822*** (0.041) | -0.810*** (0.042) |
| Guinea | -0.298*** (0.041) | -0.300*** (0.041) | -0.302*** (0.041) | -0.311*** (0.041) | -0.292*** (0.041) |
| Guyana | -0.035 (0.052) | -0.045 (0.052) | -0.032 (0.052) | -0.049 (0.052) | -0.029 (0.052) |
| Haiti | -0.330*** (0.037) | -0.331*** (0.037) | -0.331*** (0.037) | -0.331*** (0.037) | -0.331*** (0.037) |
| Honduras | -0.242*** (0.036) | -0.241*** (0.036) | -0.246*** (0.036) | -0.248*** (0.036) | -0.240*** (0.036) |
| Jordan | 0.088** (0.036) | 0.092** (0.036) | 0.090** (0.036) | 0.086** (0.036) | 0.095*** (0.036) |
| Kenya | -0.518*** (0.039) | -0.518*** (0.039) | -0.530*** (0.039) | -0.545*** (0.039) | -0.519*** (0.039) |
| Kyrgyzstan | -0.340*** (0.044) | -0.344*** (0.044) | -0.342*** (0.045) | -0.351*** (0.044) | -0.345*** (0.044) |
| Lesotho | -0.820*** (0.043) | -0.814*** (0.043) | -0.816*** (0.043) | -0.818*** (0.043) | -0.810*** (0.043) |
| Liberia | -0.392*** (0.042) | -0.398*** (0.042) | -0.389*** (0.042) | -0.399*** (0.042) | -0.384*** (0.042) |
| Madagascar | -0.759*** (0.055) | -0.784*** (0.055) | -0.764*** (0.055) | -0.792*** (0.055) | -0.783*** (0.055) |
| Mali | -0.486*** (0.039) | -0.493*** (0.039) | -0.491*** (0.039) | -0.494*** (0.039) | -0.492*** (0.039) |
| Moldova | 0.131** (0.055) | 0.135** (0.055) | 0.131** (0.055) | 0.129** (0.055) | 0.135** (0.055) |
| Morocco | 0.019 (0.048) | 0.023 (0.047) | 0.019 (0.047) | 0.015 (0.048) | 0.030 (0.048) |
| Mozambique | -0.688*** (0.039) | -0.679*** (0.039) | -0.685*** (0.039) | -0.687*** (0.039) | -0.678*** (0.039) |
| Myanmar | -0.435*** (0.045) | -0.445*** (0.045) | -0.438*** (0.045) | -0.446*** (0.045) | -0.447*** (0.045) |
| Namibia | -0.579*** (0.044) | -0.577*** (0.044) | -0.576*** (0.044) | -0.583*** (0.044) | -0.571*** (0.044) |
| Nepal | -0.587*** (0.039) | -0.582*** (0.039) | -0.589*** (0.039) | -0.595*** (0.039) | -0.585*** (0.039) |
| Niger | -0.906*** (0.043) | -0.908*** (0.043) | -0.908*** (0.043) | -0.908*** (0.043) | -0.908*** (0.043) |
| Nigeria | -0.553*** (0.036) | -0.545*** (0.037) | -0.559*** (0.037) | -0.583*** (0.036) | -0.550*** (0.037) |
| Pakistan | -0.687*** (0.041) | -0.679*** (0.042) | -0.691*** (0.042) | -0.693*** (0.041) | -0.689*** (0.042) |
| Peru | -0.314*** (0.034) | -0.313*** (0.034) | -0.308*** (0.034) | -0.315*** (0.034) | -0.315*** (0.034) |
| Rwanda | -0.873*** (0.038) | -0.879*** (0.038) | -0.873*** (0.038) | -0.881*** (0.038) | -0.872*** (0.038) |
| Senegal | -0.329*** (0.042) | -0.325*** (0.042) | -0.324*** (0.042) | -0.331*** (0.042) | -0.324*** (0.042) |
| Sierra Leone | -0.362*** (0.047) | -0.360*** (0.047) | -0.358*** (0.047) | -0.369*** (0.047) | -0.354*** (0.047) |
| South Africa | -0.524*** (0.073) | -0.527*** (0.073) | -0.520*** (0.073) | -0.533*** (0.073) | -0.525*** (0.073) |
| Tajikistan | -0.384*** (0.037) | -0.396*** (0.037) | -0.383*** (0.037) | -0.384*** (0.037) | -0.404*** (0.037) |
| Tanzania | -0.701*** (0.038) | -0.697*** (0.038) | -0.698*** (0.038) | -0.703*** (0.038) | -0.694*** (0.038) |
| Togo | -0.594*** (0.044) | -0.589*** (0.044) | -0.590*** (0.044) | -0.596*** (0.044) | -0.582*** (0.044) |
| Turkey | 0.247*** (0.043) | 0.253*** (0.043) | 0.246*** (0.043) | 0.243*** (0.043) | 0.244*** (0.043) |
| Uganda | -0.509*** (0.040) | -0.510*** (0.040) | -0.509*** (0.040) | -0.515*** (0.040) | -0.507*** (0.040) |
| Yemen | -0.774*** (0.043) | -0.786*** (0.043) | -0.779*** (0.043) | -0.791*** (0.043) | -0.786*** (0.043) |
| Zambia | -0.807*** (0.040) | -0.803*** (0.040) | -0.802*** (0.040) | -0.811*** (0.040) | -0.797*** (0.040) |
| Zimbabwe | -0.733*** (0.038) | -0.738*** (0.038) | -0.727*** (0.038) | -0.745*** (0.038) | -0.727*** (0.039) |
| 2004 | 0.021 (0.017) | 0.027 (0.017) | 0.019 (0.017) | 0.023 (0.017) | 0.026 (0.017) |
| 2005 | 0.027 (0.018) | 0.034* (0.018) | 0.031* (0.018) | 0.031* (0.018) | 0.035** (0.018) |
| 2006 | 0.008 (0.020) | 0.019 (0.020) | 0.013 (0.020) | 0.012 (0.020) | 0.020 (0.020) |
| 2007 | 0.104*** (0.019) | 0.109*** (0.019) | 0.102*** (0.019) | 0.106*** (0.019) | 0.107*** (0.019) |
| 2008 | 0.114*** (0.017) | 0.118*** (0.017) | 0.113*** (0.017) | 0.113*** (0.017) | 0.119*** (0.017) |
| 2009 | 0.136*** (0.018) | 0.140*** (0.018) | 0.137*** (0.018) | 0.135*** (0.018) | 0.143*** (0.018) |
| 2010 | 0.133*** (0.016) | 0.136*** (0.016) | 0.129*** (0.016) | 0.133*** (0.016) | 0.138*** (0.016) |
| 2011 | 0.171*** (0.016) | 0.173*** (0.016) | 0.172*** (0.016) | 0.171*** (0.016) | 0.177*** (0.016) |
| 2012 | 0.185*** (0.017) | 0.187*** (0.017) | 0.188*** (0.017) | 0.185*** (0.017) | 0.191*** (0.017) |
| 2013 | 0.225*** (0.020) | 0.235*** (0.020) | 0.225*** (0.020) | 0.237*** (0.020) | 0.230*** (0.020) |
| 2014 | 0.322*** (0.018) | 0.323*** (0.018) | 0.320*** (0.018) | 0.319*** (0.018) | 0.320*** (0.018) |
| 2015 | 0.216*** (0.020) | 0.221*** (0.020) | 0.218*** (0.020) | 0.219*** (0.020) | 0.220*** (0.020) |
| 2016 | 0.222*** (0.022) | 0.229*** (0.022) | 0.224*** (0.022) | 0.228*** (0.022) | 0.231*** (0.022) |
| 2017 | 0.299*** (0.024) | 0.310*** (0.024) | 0.302*** (0.024) | 0.299*** (0.024) | 0.318*** (0.024) |
| 2018 | 0.118*** (0.021) | 0.127*** (0.021) | 0.128*** (0.021) | 0.125*** (0.021) | 0.122*** (0.021) |
| Constant | -7.801*** (0.088) | -7.817*** (0.088) | -7.809*** (0.088) | -7.798*** (0.088) | -7.813*** (0.088) |
| Observations | 590488 | 590488 | 590488 | 590488 | 590488 |

Notes: OLS coefficients are reported. Standard errors, in parentheses, are clustered at the district level (using cluster ID). * p<0.1, ** p<0.05, *** p<0.01.

Table S4: Robustness test: Controlling for religion

|  |  |  |  | Explanatory conflict variable | | | | |
| --- | --- | --- | --- | --- | --- | --- | --- | --- |
| Dependent variable |  | (1)  Conflict1 | (2)  Conflict2 | (3)  Conflict3_  cat1 | (4)  Conflict3_  cat2 | (5)  Conflict3_  cat3 | (6)  Conflict4 | (7)  Conflict5 |
| HAZ | Coefficient | -0.013 (0.010) | -0.015*** (0.003) | -0.019 (0.013) | 0.030** (0.013) | -0.063*** (0.014) | -0.031*** (0.010) | -0.027*** (0.010) |
| WAZ | Coefficient | -0.052*** (0.007) | -0.005** (0.002) | -0.052*** (0.010) | -0.022** (0.009) | -0.094*** (0.010) | -0.028*** (0.008) | -0.009 (0.008) |
| Stunting  (HAZ<-2) | Marginal effect | 0.012*** (0.003) | 0.005*** (0.001) | 0.015*** (0.004) | 0.002 (0.004) | 0.026*** (0.004) | 0.014*** (0.003) | 0.011*** (0.003) |
| Underweight (WAZ<-2) | Marginal effect | 0.013*** (0.002) | 0.003*** (0.001) | 0.013*** (0.003) | 0.007** (0.003) | 0.021*** (0.003) | 0.005** (0.002) | 0.003 (0.002) |
| Immunization | Marginal effect | -0.029*** (0.003) | -0.007*** (0.001) | -0.015*** (0.004) | -0.033*** (0.004) | -0.044*** (0.004) | -0.028*** (0.003) | 0.008** (0.003) |
| Household has a major religion |  | Yes | Yes | Yes | Yes | Yes | Yes | Yes |
| Percentage of households with major religion at the provincial level |  | Yes | Yes | Yes | Yes | Yes | Yes | Yes |
| Control variables |  | Yes | Yes | Yes | Yes | Yes | Yes | Yes |
| Country dummies |  | Yes | Yes | Yes | Yes | Yes | Yes | Yes |
| Year dummies |  | Yes | Yes | Yes | Yes | Yes | Yes | Yes |
| Observations |  | 406967 | 406967 | 406967 | 406967 | 406967 | 406967 | 406967 |

Notes: OLS coefficients and Probit marginal effects are reported. Columns (3)-(5) refer to coefficients/marginal effects from the same regression where Conflict3_cat1-Conflict3_cat3 are jointly included as controls. Other control variables as in Table 2. Standard errors in parentheses clustered at district level (using cluster ID). * p<0.1, ** p<0.05, *** p<0.01.

Table S5: Robustness test: controlling for a proxy of provincial population

|  | (1) | (2) | (3) | (4) | (5) |
| --- | --- | --- | --- | --- | --- |
|  | HAZ  [OLS] | WAZ  [OLS] | Stunting  [Probit] | Underweight  [Probit] | Immunization  [Probit] |
| *Panel A: Include all children* | | | | | |
| Reference category: Children not exposed to conflict (Conflict1 = 0) | | | | | |
| Conflict1 | -0.053*** (0.007) | -0.078*** (0.006) | 0.021*** (0.002) | 0.011*** (0.002) | -0.012*** (0.003) |
| Estimated population at the provincial level | Yes | Yes | Yes | Yes | Yes |
| Control variables | Yes | Yes | Yes | Yes | Yes |
| Country dummies | Yes | Yes | Yes | Yes | Yes |
| Adjusted *R*^2^ | 0.20 | 0.22 |  |  |  |
| Observations | 590488 | 590488 | 590488 | 590488 | 590488 |
| *Panel B: Include only children exposed to conflict* | | | | | |
| Reference category: Children exposed to conflict with no death (Conflict3 = 0) | | | | | |
| Conflict3_cat1 | -0.104*** (0.022) | -0.109*** (0.016) | 0.042*** (0.007) | 0.029*** (0.005) | 0.010 (0.008) |
| Conflict3_cat2 | -0.038* (0.022) | -0.074*** (0.016) | 0.022*** (0.007) | 0.020*** (0.005) | -0.006 (0.008) |
| Conflict3_cat3 | -0.054** (0.023) | -0.088*** (0.017) | 0.031*** (0.007) | 0.022*** (0.005) | 0.003 (0.008) |
| Estimated population at the provincial level | Yes | Yes | Yes | Yes | Yes |
| Control variables | Yes | Yes | Yes | Yes | Yes |
| Country dummies | Yes | Yes | Yes | Yes | Yes |
| Adjusted *R*^2^ | 0.19 | 0.22 |  |  |  |
| Observations | 240832 | 240832 | 240832 | 240832 | 240832 |

Notes: Other control variables include those in Table 2. Coefficients are reported in columns 1 and 2, marginal effects are reported in columns 3, 4 and 5. Standard errors in parentheses are clustered at the district level (using cluster ID). * p<0.1, ** p<0.05, *** p<0.01.

Table S6: Results including India

|  | (1) | (2) | (3) | (4) | (5) |
| --- | --- | --- | --- | --- | --- |
|  | HAZ  [OLS] | WAZ  [OLS] | Stunting  [Probit] | Underweight  [Probit] | Immunization  [Probit] |
|  |  |  |  |  |  |
| *Sample: Children exposed to conflict* | | | | | |
| Reference category: Children exposed to conflict with no death (Conflict3 = 0) | | | | | |
| Conflict3_cat1 | -0.022 (0.028) | -0.088*** (0.024) | 0.030*** (0.009) | 0.031*** (0.009) | -0.084*** (0.011) |
| Conflict3_cat2 | 0.021 (0.029) | -0.146*** (0.024) | 0.022** (0.010) | 0.049*** (0.009) | -0.091*** (0.011) |
| Conflict3_cat3 | 0.004 (0.029) | -0.080*** (0.025) | 0.027*** (0.010) | 0.027*** (0.009) | -0.073*** (0.011) |
| Estimated population at the provincial level | Yes | Yes | Yes | Yes | Yes |
| Control variables | Yes | Yes | Yes | Yes | Yes |
| Country dummies | Yes | Yes | Yes | Yes | Yes |
| Adjusted *R*^2^ | 0.18 | 0.22 |  |  |  |
| Observations | 422937 | 422937 | 422937 | 422937 | 422937 |

Notes: Other control variables include those in Table 2. Coefficients are reported in columns 1 and 2, marginal effects are reported in columns 3 and 4. Standard errors in parentheses are clustered at the district level (using cluster ID). De-normalized women sampling weights are used in regressions. * p<0.1, ** p<0.05, *** p<0.01.

Table S7: Results disaggregated by world regions

|  | (1) | (2) | (3) | (4) | (5) |
| --- | --- | --- | --- | --- | --- |
|  | HAZ  [OLS] | WAZ  [OLS] | Stunting  [Probit] | Underweight  [Probit] | Immunization  [Probit] |
| Reference category: Children not exposed to conflict (Conflict1 = 0) | | | | | |
| *Panel A: Africa* | | | | | |
| Conflict1 | -0.020* (0.012) | -0.035*** (0.008) | 0.017*** (0.003) | 0.008*** (0.002) | -0.034*** (0.004) |
| Control variables | Yes | Yes | Yes | Yes | Yes |
| Country dummies | Yes | Yes | Yes | Yes | Yes |
| Adjusted *R*^2^ | 0.17 | 0.16 |  |  |  |
| Observations | 357161 | 357161 | 357161 | 357161 | 357161 |
| *Panel B: Asia* | | | | | |
| Conflict1 | -0.053** (0.022) | -0.047*** (0.017) | 0.014** (0.007) | 0.005 (0.006) | 0.029*** (0.007) |
| Control variables | Yes | Yes | Yes | Yes | Yes |
| Country dummies | Yes | Yes | Yes | Yes | Yes |
| Adjusted *R*^2^ | 0.25 | 0.29 |  |  |  |
| Observations | 68531 | 68531 | 68531 | 68531 | 68531 |
| *Panel C: Europe* | | | | | |
| Conflict1 | -0.044 (0.061) | -0.116** (0.051) | 0.015 (0.017) | -0.000 (0.010) | -0.077*** (0.020) |
| Control variables | Yes | Yes | Yes | Yes | Yes |
| Country dummies | Yes | Yes | Yes | Yes | Yes |
| Adjusted *R*^2^ | 0.13 | 0.10 |  |  |  |
| Observations | 9981 | 9981 | 9981 | 9981 | 9981 |
| *Panel D: America* | | | | | |
| Conflict1 | -0.083*** (0.010) | -0.131*** (0.009) | 0.020*** (0.004) | 0.009*** (0.002) | 0.012*** (0.004) |
| Control variables | Yes | Yes | Yes | Yes | Yes |
| Country dummies | Yes | Yes | Yes | Yes | Yes |
| Adjusted *R*^2^ | 0.31 | 0.19 |  |  |  |
| Observations | 126670 | 126670 | 126670 | 126670 | 126670 |
| *Panel E: Middle East* | | | | | |
| Conflict1 | -0.057 (0.039) | -0.053* (0.028) | 0.006 (0.009) | 0.019*** (0.007) | -0.068*** (0.013) |
| Control variables | Yes | Yes | Yes | Yes | Yes |
| Country dummies | Yes | Yes | Yes | Yes | Yes |
| Adjusted *R*^2^ | 0.28 | 0.38 |  |  |  |
| Observations | 28145 | 28145 | 28145 | 28145 | 28145 |

Notes: Other control variables include those in Table 2. Coefficients are reported in columns 1 and 2, marginal effects are reported in columns 3 and 4. Standard errors in parentheses are clustered at the district level (using cluster ID). * p<0.1, ** p<0.05, *** p<0.01.

Table S8: Results disaggregated by rural/urban residence

|  | (1) | (2) | (3) | (4) | (5) |
| --- | --- | --- | --- | --- | --- |
|  | HAZ  [OLS] | WAZ  [OLS] | Stunting  [Probit] | Underweight  [Probit] | Immunization  [Probit] |
| Reference category: Children not exposed to conflict (Conflict1 = 0) | | | | | |
| *Panel A: Rural areas* | | | | | |
| Conflict1 | -0.024** (0.010) | -0.039*** (0.007) | 0.013*** (0.003) | 0.006*** (0.002) | -0.012*** (0.003) |
| Control variables | Yes | Yes | Yes | Yes | Yes |
| Country dummies | Yes | Yes | Yes | Yes | Yes |
| Adjusted *R*^2^ | 0.19 | 0.19 |  |  |  |
| Observations | 382181 | 382181 | 382181 | 382181 | 382181 |
| *Panel B: Urban areas* | | | | | |
| Conflict1 | -0.058*** (0.011) | -0.105*** (0.009) | 0.021*** (0.003) | 0.013*** (0.002) | -0.017*** (0.004) |
| Control variables | Yes | Yes | Yes | Yes | Yes |
| Country dummies | Yes | Yes | Yes | Yes | Yes |
| Adjusted *R*^2^ | 0.17 | 0.20 |  |  |  |
| Observations | 208307 | 208307 | 208307 | 208307 | 208307 |

Notes: Other control variables include those in Table 2. Coefficients are reported in columns 1 and 2, marginal effects are reported in columns 3 and 4. Standard errors in parentheses are clustered at the district level (using cluster ID). * p<0.1, ** p<0.05, *** p<0.01.

Table S9: Results disaggregated by household wealth quintiles

|  | (1) | (2) | (3) | (4) | (5) |
| --- | --- | --- | --- | --- | --- |
|  | HAZ  [OLS] | WAZ  [OLS] | Stunting  [Probit] | Underweight  [Probit] | Immunization  [Probit] |
| Reference category: Children not exposed to conflict (Conflict1 = 0) | | | | | |
| *Panel A: Wealth quintile- Poorest* | | | | | |
| Conflict1 | -0.024* (0.014) | -0.031*** (0.010) | 0.009** (0.004) | 0.004 (0.004) | -0.010** (0.005) |
| Control variables | Yes | Yes | Yes | Yes | Yes |
| Country dummies | Yes | Yes | Yes | Yes | Yes |
| Adjusted *R*^2^ | 0.19 | 0.20 |  |  |  |
| Observations | 147668 | 147668 | 147668 | 147668 | 147668 |
| *Panel B: Wealth quintile- Poorer* | | | | | |
| Conflict1 | -0.048*** (0.013) | -0.064*** (0.010) | 0.018*** (0.004) | 0.009*** (0.003) | -0.008* (0.004) |
| Control variables | Yes | Yes | Yes | Yes | Yes |
| Country dummies | Yes | Yes | Yes | Yes | Yes |
| Adjusted *R*^2^ | 0.19 | 0.21 |  |  |  |
| Observations | 131503 | 131503 | 131503 | 131503 | 131503 |
| *Panel C: Wealth quintile- Middle* | | | | | |
| Conflict1 | -0.052*** (0.014) | -0.070*** (0.011) | 0.026*** (0.005) | 0.011*** (0.003) | -0.012** (0.005) |
| Control variables | Yes | Yes | Yes | Yes | Yes |
| Country dummies | Yes | Yes | Yes | Yes | Yes |
| Adjusted *R*^2^ | 0.19 | 0.22 |  |  |  |
| Observations | 118692 | 118692 | 118692 | 118692 | 118692 |
| *Panel D: Wealth quintile- Richer* | | | | | |
| Conflict1 | -0.018 (0.016) | -0.090*** (0.012) | 0.014*** (0.005) | 0.012*** (0.003) | -0.027*** (0.005) |
| Control variables | Yes | Yes | Yes | Yes | Yes |
| Country dummies | Yes | Yes | Yes | Yes | Yes |
| Adjusted *R*^2^ | 0.17 | 0.22 |  |  |  |
| Observations | 104339 | 104339 | 104339 | 104339 | 104339 |
| *Panel E: Wealth quintile- Richest* | | | | | |
| Conflict1 | -0.028 (0.019) | -0.060*** (0.014) | 0.015*** (0.005) | 0.011*** (0.003) | -0.017*** (0.005) |
| Control variables | Yes | Yes | Yes | Yes | Yes |
| Country dummies | Yes | Yes | Yes | Yes | Yes |
| Adjusted *R*^2^ | 0.14 | 0.19 |  |  |  |
| Observations | 88286 | 88286 | 88286 | 88286 | 88286 |

Notes: Other control variables include those in Table 2. Coefficients are reported in columns 1 and 2, marginal effects are reported in columns 3 and 4. Standard errors in parentheses are clustered at the district level (using cluster ID). * p<0.1, ** p<0.05, *** p<0.01.

Table S10: The association between conflict intensity and child health outcomes

|  | (1) | (2) | (3) | (4) | (5) |
| --- | --- | --- | --- | --- | --- |
|  | HAZ  [OLS] | WAZ  [OLS] | Stunting  [Probit] | Underweight  [Probit] | Immunization  [Probit] |
| *Panel A: With country fixed effects* | | | | | |
| Square root of death per year of conflict exposure per capita | -0.029 (0.029) | -0.088*** (0.022) | 0.039*** (0.007) | 0.006 (0.006) | -0.071*** (0.010) |
| Control variables | Yes | Yes | Yes | Yes | Yes |
| Adjusted *R*^2^ | 0.20 | 0.22 |  |  |  |
| *Panel B: With province fixed effects* | | | | | |
| Square root of death per year of conflict exposure per capita | 0.014 (0.037) | -0.044* (0.026) | 0.018** (0.009) | 0.010 (0.007) | -0.141*** (0.012) |
| Control variables | Yes | Yes | Yes | Yes | Yes |
| Adjusted *R*^2^ | 0.21 | 0.23 |  |  |  |
| Observations | 590488 | 590488 | 590488 | 590488 | 590488 |

Notes: Other control variables include those in Table 2. Coefficients are reported in columns 1 and 2, marginal effects are reported in columns 3, 4 and 5. Standard errors in parentheses are clustered at the district level (using cluster ID). * p<0.1, ** p<0.05, *** p<0.01.

**Supplementary figures**

Figure S1: Mean of provinces’ estimated population by country


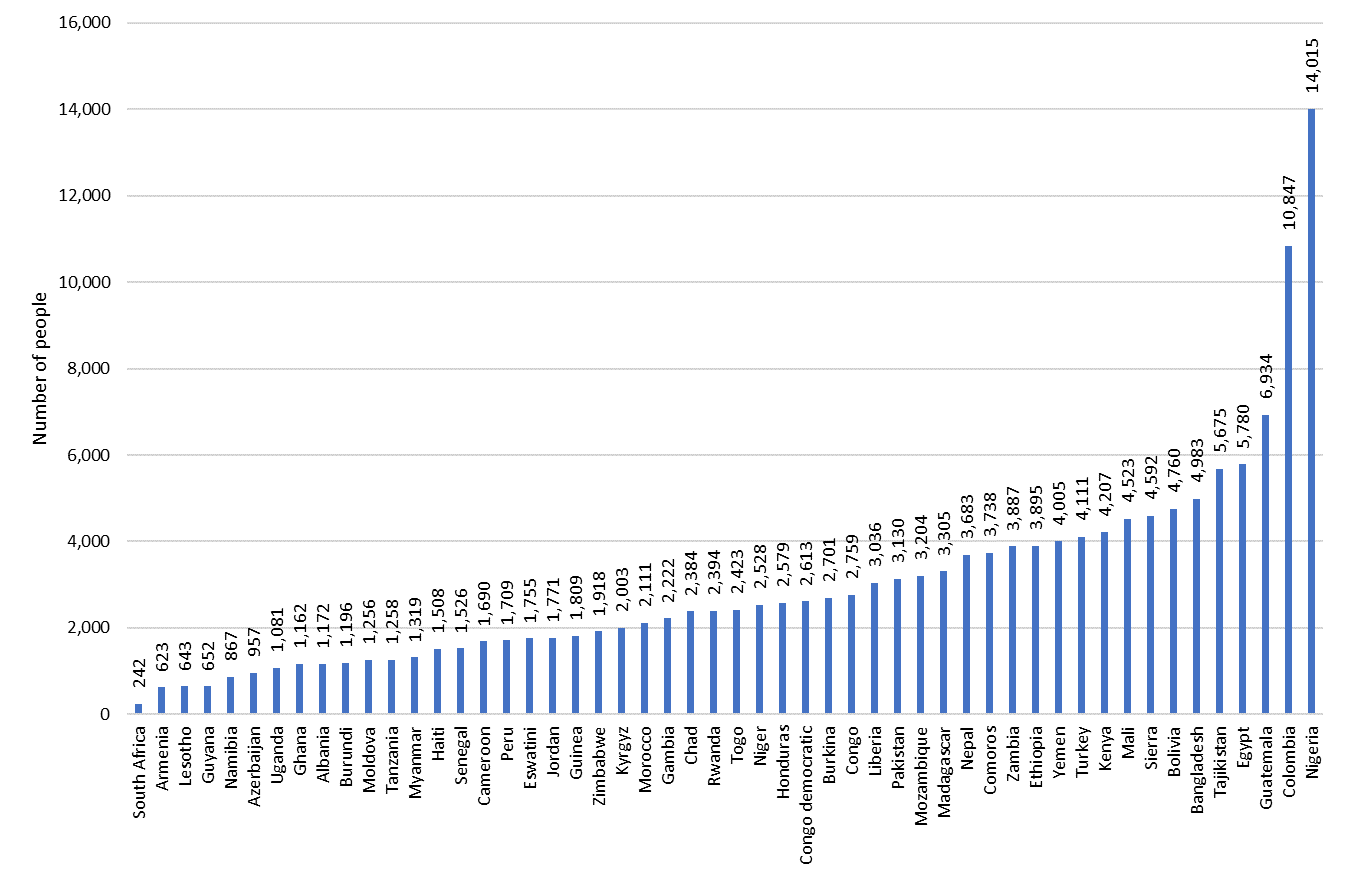

Supplement: Supplementary file 1 — Additional file 1: Table S1. Sample characteristics by country. Table S2: Conflict status during 1997 to 2018 by country. Table S3: OLS estimates, full set of results - dependent variable: HAZ. Table S4: Robustness test: Controlling for religion. Table S5: Robustness test: controlling for a proxy of provincial population. Table S6: Results including India. Table S7: Results disaggregated by world regions. Table S8: Results disaggregated by rural/urban residence. Table S9: Results disaggregated by household wealth quintiles. Table S10: The association between conflict intensity and child health outcomes. Fig. S1. Mean of provinces’ estimated population by country [file 13031_2022_483_MOESM1_ESM.docx]
